# Spatial organization of intratumoral microbiome and regional tumor immunity

**DOI:** 10.3389/fimmu.2026.1961794

**Published:** 2026-10-05

**Authors:** Jhommara Bautista, Andrés López-Cortés

**Affiliations:** Cancer Research Group (CRG), Faculty of Medicine, Universidad de Las Américas, Quito, Ecuador

**Keywords:** immune ecotypes, intratumoral microbiome, microbial microniches, spatial immunology, tumor immune microenvironment

## Abstract

Solid tumors are spatially heterogeneous ecosystems in which immune recruitment, anatomical access, functional competence, stromal organization, vascularization and physicochemical conditions vary across regions. The conventional “hot–cold” dichotomy therefore obscures the coexistence of immune-inflamed, immune-excluded, immune-suppressed and immune-desert territories within the same lesion. Intratumoral microorganisms add a second layer of heterogeneity, yet microbial signals are often interpreted from bulk abundance without resolving their location, host-cell compartment, viability or functional state. The distinct contribution of this review is the integration of spatial microbial signals with regional immune ecotypes defined independently from microbial presence. Immune-inflamed, immune-excluded, immune-desert, and immune-suppressed regions are assigned from host features of immune recruitment, access to malignant cells, functional competence, and multicellular organization before microbial localization, cellular compartmentalization, and biological state are considered. We examine how intracellular microbial material in malignant cells, phagocyte-associated signals, extracellular biofilms and bacteria-positive microniches may differentially engage HLA-mediated antigen presentation, cGAS–STING or direct STING signaling, TLR–MyD88–TRAF6 pathways, dendritic-cell and natural-killer-cell circuits, and myeloid, stromal and metabolic programs. Depending on microbial identity, burden, persistence and cellular address, these interactions may support cytotoxic immunity or promote chronic inflammation, suppressive myeloid accumulation, neutrophil extracellular traps, regulatory T-cell expansion, T-cell dysfunction and matrix-associated exclusion. We further assess physicochemical selection, microbial metabolism, metastatic persistence, treatment-associated remodeling and spatial multi-omic strategies for defining clinically relevant immunomicrobial niches. Current evidence remains limited by low biomass, contamination risk, incomplete source attribution, cross-sectional sampling and scarce causal validation. Future studies should combine multiregional and longitudinal sampling with orthogonal microbial detection, cell-resolved host profiling, viability and activity measurements, and controlled perturbation. This model reframes tumors as mosaics of regional immune ecotypes in which spatially localized microbial signals acquire context-dependent biological meaning.

## Introduction

The immune microenvironment of a solid tumor is not a single state but a mosaic of regional ecosystems. The conventional labels of immunologically ‘hot’ and ‘cold’ remain useful shorthand for broad differences in immune infiltration, but they compress several non-equivalent variables, including immune-cell abundance, composition, anatomical access, functional competence and multicellular organization, into a single tumor-level descriptor (1, 2). Lymphocytes may penetrate malignant-cell nests, accumulate at the invasive margin or within the stroma without effective tumor contact, remain scarce because recruitment is limited, or be numerically abundant yet functionally constrained. Established tumor-level classifications distinguish inflamed, immune-altered, and cold phenotypes according to immune recruitment, localization, and functional state (1, 3). Spatial studies further show that distinct immune architectures and cellular neighborhoods can coexist within the same lesion (2, 4). For the regional analysis used here, these patterns are operationalized as immune-inflamed, immune-excluded, immune-desert, and immune-suppressed ecotypes.

The biological importance of immune localization was established by studies showing that the type, density and distribution of immune cells across the tumor center and invasive margin provide information beyond total immune abundance. In colorectal cancer (CRC), their combined assessment predicted recurrence and survival more effectively than conventional histopathological staging alone (5). Pan-cancer immunogenomic analyses subsequently identified immune subtypes that differed in lymphocyte and macrophage signatures, T-helper-cell orientation, proliferative activity, immunomodulatory programs and clinical outcome. Notably, the subtype with the greatest lymphocytic infiltration was not uniformly associated with the most favorable biology, demonstrating that the functional and compositional context of inflammation matters as much as its magnitude. These immune subtypes were derived largely from bulk molecular profiling; the analysis did not resolve whether contrasting immune programs occupied the same or different regions within individual tumors (6).

Spatially resolved analyses have revealed marked variation in tumor immune architecture. Multiplexed imaging of triple-negative breast cancer distinguished immune-poor, mixed, and compartmentalized patterns and showed that tumors with similar conventional lymphocyte scores could differ substantially in immune–malignant cell contact. The cellular sources of PD-1, PD-L1, and IDO also varied according to tissue architecture, indicating regulatory configurations that could not be inferred from marker abundance alone (2). In CRC, highly multiplexed imaging identified conserved cellular neighborhoods whose coupling, fragmentation, and communication at the invasive front were associated with clinical outcome (4). Single-cell and spatial transcriptomic analyses also identified localized multicellular immune hubs composed of coordinated malignant, myeloid, and lymphoid programs. Whole-tumor immune labels therefore provide limited information about regional states defined by immune recruitment, access to malignant cells, functional state, and cellular interaction topology (7).

The intratumoral microbiome adds another source of spatial heterogeneity. Microbial DNA, RNA, structural components, and intact microbial forms have been detected across several human tumor types, with bacterial signals found predominantly in malignant and immune cells. Detection within defined cellular compartments, however, does not indicate that every microbial sequence derives from an intact, viable, or metabolically active microorganism (8, 9).

Interpretation of the intratumoral microbiome requires explicit distinction among different forms of microbial evidence. A tumor-associated signal may derive from an intact microorganism, a nonviable but structurally preserved cell, extracellular microbial material, or products that have been internalized and processed by host cells. DNA sequencing establishes the presence of microbial genetic material but cannot independently determine viability, cellular localization, or stable residence, particularly in low-biomass tumors in which contamination control is critical. RNA detection provides stronger support for transcriptionally preserved or recently viable microorganisms, although it does not by itself demonstrate replication or cultivability. Microbial peptides and structural products provide evidence of microbial exposure or processing; HLA-bound microbial peptides further demonstrate entry of microbial protein material into host antigen-presentation pathways, but neither observation establishes that the source microorganism remains viable or metabolically active at the time of sampling. Evidence for active microbial residence is strongest when spatial localization is supported by intact morphology, microbial transcription, cultivation, replication, or directly measured function (8, 10, 11).

Spatial studies further indicate that microbial signals are unevenly distributed within tumors. In oral squamous cell carcinoma (OSCC) and CRC, bacteria-positive microniches occurred beside bacteria-negative regions and coincided with distinct local epithelial and immune states. Their cross-sectional characterization, however, cannot determine whether microorganisms generated these environments, preferentially occupied pre-existing niches, or participated in reciprocal ecological selection (12).

The central unresolved question is consequently not simply which microorganisms are detectable in tumors, but where microbial material is located, which host or extracellular compartments contain or encounter it and what biological state the detected signal represents. Tumor-wide abundance cannot distinguish a signal arising from a mucosal biofilm from intracellular bacterial material in malignant cells, microbial material retained by phagocytic myeloid cells or a focal population within a poorly vascularized microniche. Resolving these configurations requires regional information on microbial distribution across the tumor center, invasive margin, stromal or vascular interfaces, and physiologically distinct territories, together with identification of the immune populations positioned nearby and their local activation, exclusion, or functional suppression states (8, 12, 13).

Metastatic disease adds another layer of spatial and temporal variation. A pan-cancer analysis of 4,160 metastatic biopsies identified organ-specific microbial tropisms, enrichment of anaerobic taxa in hypoxic tumors, associations between microbial diversity and tumor-infiltrating neutrophils, and temporal changes in microbial communities across longitudinal biopsies obtained during systemic treatment (14). Such associations do not by themselves establish microbial viability, local activity, causality, or the direction of host–microbial interactions. Matched primary colorectal tumors and liver metastases, together with experimental breast cancer models, indicate that selected bacteria can persist during metastatic dissemination, while community composition may also be altered by the metastatic organ and treatment (10, 14, 15). Distinguishing persistence from site- or therapy-driven remodeling will require paired spatial and functional analyses rather than taxonomic comparison alone.

Recent reviews focused specifically on the spatial organization of the intratumoral microbiome have described regional microbial heterogeneity, compartment-specific localization, and the need to distinguish sequencing-based detection from tissue-localized and mechanistically supported observations (16–18). These reviews organize current evidence around microbial distribution, spatial resolution, methodological approaches, and strength of biological support. The present review uses a different organizing unit: regional immune ecotypes are defined independently from microbial detection, and microbial localization, cellular compartmentalization, and biological state are subsequently interpreted within those host-defined regions. Spatial heterogeneity and evidence stratification are therefore treated as established interpretive principles rather than novel concepts.

Spatial evidence is interpreted according to what can be localized within the tumor. Microbial signals detected together with host-cell or immune features in the same tissue region provide evidence of regional microbial–immune organization. Assignment of microbial material to malignant cells, macrophages, or other cellular populations establishes cellular compartmentalization but does not preserve the anatomical relationships among those populations. Tumor-wide microbial abundance or transcriptional associations can identify relationships with immune composition or activity, but they cannot determine whether microbial and immune signals occupy the same region. Functional perturbation can establish that a microorganism or microbial product alters a defined host pathway, yet it does not demonstrate that the interaction occurs in the corresponding human tumor microniche. Regional localization, cellular compartmentalization, tumor-level association, and functional causality are therefore considered separately throughout the review (8, 12).

The organizing principle of this review is the regional immune ecotype rather than microbial abundance or taxonomic composition. Immune-inflamed, immune-excluded, immune-desert, and immune-suppressed regions are defined independently from microbial detection using host spatial features of immune recruitment, access to malignant cells, functional competence, and multicellular organization. Microbial signals are subsequently examined within those predefined regions according to their anatomical localization, host-cell compartment, and biological state. This separation avoids assigning an immune phenotype from the same microbial feature subsequently used to interpret that phenotype. Human tumor observations provide the primary basis for spatial interpretation, while experimental evidence is used to assess mechanisms capable of explaining the regional immune configurations observed or proposed in tumor tissue. Cross-cancer comparisons are used to determine which immunomicrobial relationships recur across solid tumors and which remain dependent on tumor anatomy, microbial identity or burden, and cellular localization. Regional immune ecotypes and their associated microbial, cellular, and physiological configurations are summarized in **Figure 1**.

**Figure 1:**
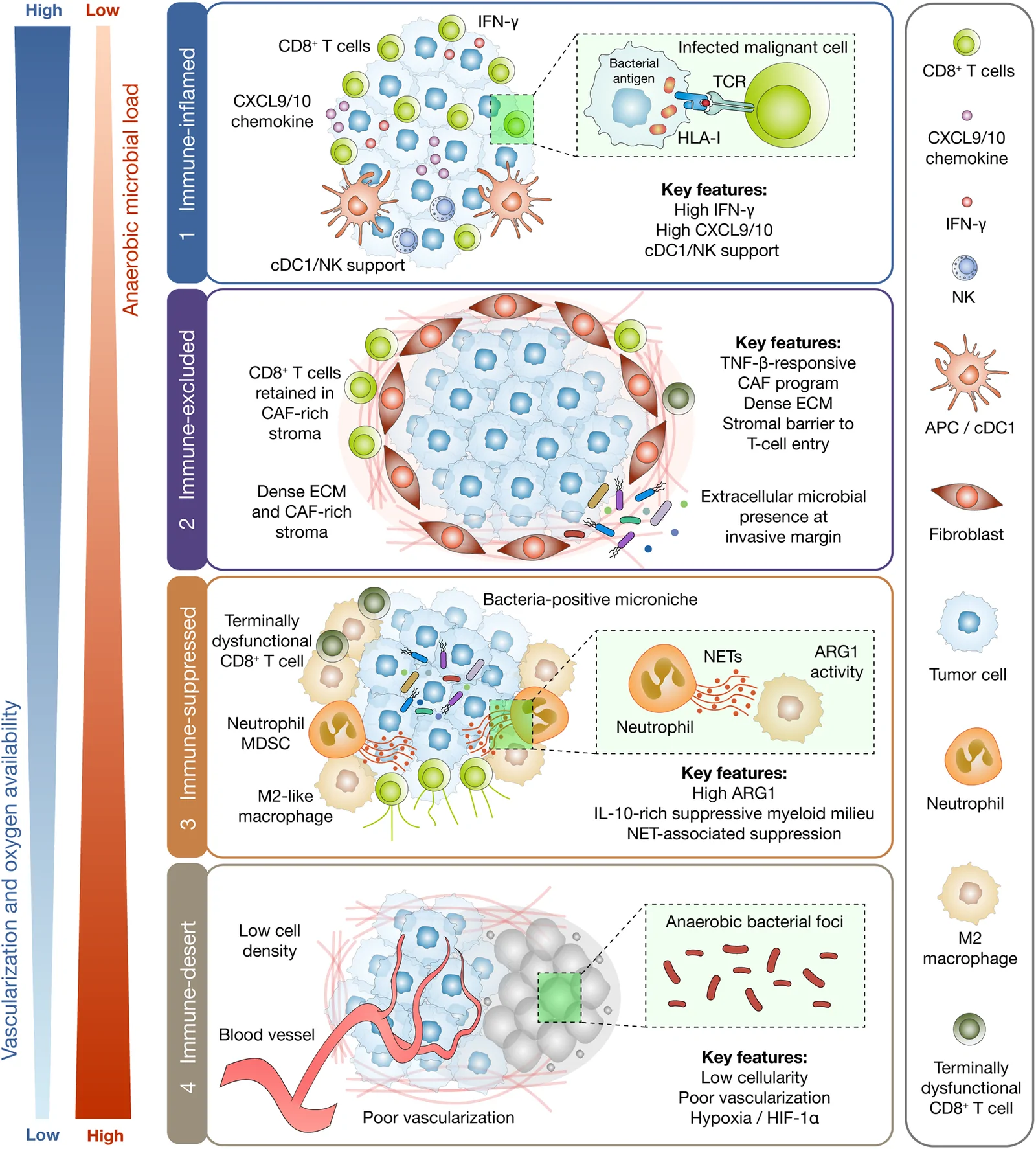
Regional immune ecotypes and illustrative microbial configurations within heterogeneous solid tumors. Conceptual cross-section illustrating the spatial coexistence of four regional immune ecotypes. The ecotypes are defined by host immune recruitment, access to malignant-cell nests, functional competence, and multicellular organization; the microbial features are superimposed subsequently and do not define ecotype assignment. Their vertical arrangement and the lateral gradients represent schematic, context-dependent tendencies rather than a measured continuum, fixed temporal sequence, universal anatomical organization, or mutually exclusive whole-tumor states. Immune-inflamed region: CD8^+^ T cells penetrate malignant-cell nests and participate in local interferon-γ (IFN-γ)–CXCL9/CXCL10 chemokine circuits supported by conventional type 1 dendritic cells (cDC1s) and natural killer (NK) cells. The magnified inset depicts a proposed route through which an infected or bacteria-exposed malignant cell processes intracellular bacterial proteins and presents bacteria-derived peptides on human leukocyte antigen class I (HLA-I) molecules for recognition by the T-cell receptor (TCR) of a CD8^+^ T cell. Immune-excluded region: recruited CD8^+^ T cells are retained at the tumor–stroma interface or within cancer-associated fibroblast (CAF)-rich stroma and show limited penetration of malignant-cell nests. A TGF-β-responsive CAF program and dense extracellular matrix may contribute to this spatial barrier. Extracellular microbial signals may occur at selected invasive or mucosal margins; however, direct evidence that intratumoral microorganisms generate a continuous CAF-dependent exclusion barrier remains limited. T-cell dysfunction may coexist with exclusion but is not required for classification as immune-excluded. Immune-suppressed region: an immune-infiltrated, bacteria-positive microniche is associated with suppressive myeloid populations, including M2-like macrophages, neutrophils, and, where functionally validated, myeloid-derived suppressor cells (MDSCs). Arginase 1 (ARG1), interleukin-10 (IL-10), neutrophil extracellular traps (NETs), and terminally dysfunctional CD8^+^ T cells illustrate non-exclusive mechanisms capable of constraining local antitumor activity. NET-associated suppression is context-dependent and should not be interpreted as a generalized physical barrier to T-cell entry. Immune-desert region: hypocellular and poorly vascularized territories display limited immune recruitment and may coincide with hypoxia, hypoxia-inducible factor 1α (HIF-1α) activity, and conditions compatible with enrichment of anaerobic microbial profiles. The depicted anaerobic bacterial focus is conceptual because tumor-level associations between hypoxia and anaerobic taxa do not demonstrate microscopic colocalization. The lateral arrows schematically indicate decreasing vascularization and oxygen availability and increasing anaerobic microbial signal from top to bottom; these tendencies are neither defining criteria for the ecotypes nor presumed to occur in every tumor. Symbols identify the principal malignant, stromal, vascular, immune, and microbial components.

## Literature search and study selection

For this narrative review, relevant literature was identified through searches of PubMed/MEDLINE, Scopus, and Web of Science Core Collection from database inception through August 2026. Search terms combined concepts related to the intratumoral microbiome (intratumoral microbiome, tumor microbiome, intratumoral bacteria, intratumoral fungi, tumor mycobiome) with terms describing spatial organization (spatial, localization, microniche, fluorescence *in situ* hybridization (FISH), RNAscope, spatial transcriptomics, multiplex imaging) and regional tumor immunity (immune microenvironment, T cell, myeloid, immune exclusion, immune suppression, immune desert). Studies were selected according to their relevance to the spatial and mechanistic questions addressed in the review, without applying formal systematic-review eligibility criteria. Primary human tumor studies were prioritized when microbial signals and host or immune features were resolved within the same tissue region or anatomically registered compartments. Studies providing cell-resolved microbial localization were distinguished from tumor-level sequencing or molecular associations. Experimental studies were prioritized when they directly tested microbial effects on immune, stromal, metabolic, or signaling processes relevant to regional configurations observed or proposed in human tumors. Reviews were used mainly for background context and identification of relevant primary literature, whereas spatial and mechanistic interpretations were based preferentially on original studies.

## Beyond the hot–cold binary and the spatial heterogeneity of tumor immune states

The conventional classification of tumors as immunologically “hot” or “cold” compresses several biologically distinct features into a single descriptor of immune-cell abundance. Immune density, cellular composition, anatomical localization, functional state, and interaction topology, however, represent different dimensions of the tumor immune microenvironment (2, 5). A tumor may contain abundant lymphocytes confined to the invasive margin or stromal compartment, whereas a lesion with a similar immune-cell burden may permit direct infiltration of malignant-cell nests (19, 20). CD8^+^ T-cell presence alone also does not indicate cytotoxic competence, productive contact with tumor cells, or adequate local support for sustained antitumor activity (2, 4). In CRC, densities of CD3^+^, CD8^+^, granzyme B^+^, and CD45RO^+^ cells measured in the tumor center and invasive margin were associated with recurrence and survival, while combined assessment of both regions provided greater prognostic information than evaluation of either region alone. Immune localization therefore became a defining component of the immune contexture rather than a secondary measure of infiltration (5).

Pan-cancer immunogenomic analyses also indicate that immune states do not follow a simple continuum from low to high infiltration. Analysis of more than 10,000 tumors across 33 cancer types identified six immune subtypes, wound healing, IFN-γ dominant, inflammatory, lymphocyte depleted, immunologically quiet, and TGF-β dominant, with differences in lymphocyte and macrophage signatures, T-helper-cell orientation, proliferative activity, immunomodulatory programs, and clinical outcome (6). High immune infiltration was not consistently associated with favorable biology, underscoring the importance of cellular composition and functional state in addition to immune-cell abundance. Because subtype assignments were derived largely from bulk molecular measurements, spatial coexistence or segregation of contrasting immune programs within individual tumors remains unresolved (2, 4, 6, 7).

Existing tumor-level immune classifications distinguish inflamed or hot, altered-excluded, altered-immunosuppressed, and cold phenotypes. For the regional spatial classification used in this review, we distinguish four immune ecotypes: immune-inflamed, immune-excluded, immune-desert, and immune-suppressed (1, 3). Each ecotype is defined by the intersection of four dimensions: immune-cell recruitment, physical access to malignant cells, functional competence and multicellular organization. Microbial presence, abundance, or identity is not used to assign an immune ecotype; microbial features are evaluated only after the regional immune state has been established from host spatial architecture and immune function. Accordingly, “immune-altered” is retained only when referring to previously established tumor-level classifications and is not used interchangeably with the regional immune-suppressed ecotype. Distinct ecotypes may occupy the invasive margin, fibrotic stroma, tumor-cell nests, perivascular domains, hypoxic or necrotic territories and organized lymphoid aggregates within the same lesion (2, 4, 7).

Immune-inflamed regions. Immune-inflamed regions are characterized by cytotoxic lymphocytes positioned within or immediately adjacent to malignant-cell nests, rather than merely accumulating in the surrounding stroma. In triple-negative breast cancer, fully inflamed tumors contained CD8^+^ T cells in both stromal and epithelial compartments and were distinguished by granzyme B expression, a type I interferon-associated signature and other features of cytotoxic activation. By contrast, tumors with substantial stromal CD8^+^ infiltration but limited epithelial penetration represented spatially restricted states. Thus, the identification of an inflamed ecotype requires evidence of intranest localization and functional activity, not CD8^+^ density alone (21).

Productive inflammation is frequently sustained by local IFN-γ–chemokine circuits. In CRC, CXCL10- and CXCL11-expressing malignant and myeloid cells formed spatial foci enriched in CD3E^+^, IFNG^+^ and CXCL13^+^ T cells. Chemokine-positive malignant cells were located closer to activated T cells than chemokine-negative cells, supporting a spatially restricted feedback circuit in which lymphocyte-derived IFN-γ induces CXCL9, CXCL10 and CXCL11, thereby promoting further recruitment of CXCR3^+^ effector populations (7). CCL5 can cooperate with these ligands by supporting the recruitment or retention of CCR5^+^ cytotoxic lymphocytes. The presence of IFN-γ-responsive signaling and coordinated CXCL9–CXCL11 and CCL5 expression therefore strengthens the assignment of an immune-inflamed ecotype, although no single chemokine is universally required.

Conventional type 1 dendritic cells (cDC1s) support inflamed regions through tumor-antigen acquisition, cross-presentation, and activation of CD8^+^ T cells. Their recruitment can be coordinated with natural killer (NK) cells, which contribute to direct cytotoxicity, early IFN-γ production, and secretion of CCL5 and XCL1. Experimental models showed that NK-cell-derived CCL5 and XCL1 promoted cDC1 accumulation within tumors, while human tumor analyses identified concordant NK-cell, cDC1, and chemokine-associated signatures. Detectable NK cells and cDC1, however, should not be considered obligatory features of every inflamed region. Their co-occurrence is consistent with a type 1 immune circuit linking innate cytotoxicity, antigen presentation, and CD8^+^ T-cell responses (22, 23).

The functional quality of inflamed regions also depends on whether the tissue contains niches capable of maintaining renewable T-cell states. In hepatocellular carcinoma, LAMP3^+^ mature dendritic cells were positioned near CXCL13^+^ helper T cells and TCF1^+^ progenitor-like CD8^+^ T cells. These populations formed spatially enriched cellular triads, and their proximity was associated with CD8^+^ differentiation toward effector-like rather than terminally dysfunctional states (24, 25). The organization was supported by multiple spatial technologies, indicating that it could not be explained solely by increased abundance of the individual populations.

Tertiary lymphoid structures (TLSs) represent a related but structurally distinct form of local immune organization. In melanoma, CD20^+^ B-cell aggregates surrounded predominantly by CD4^+^ T cells expressed CXCL13-, CXCR5- and DC-LAMP-associated features. TLS-containing tumors were accompanied by tumor-associated CD8^+^ T cells and contained greater proportions of TCF7^+^ naive- or memory-like lymphocytes, whereas T cells outside TLS-supported environments displayed more dysfunctional phenotypes (26).

TLSs should not be conflated with all CXCL13-rich immune aggregates. Chemokine-organized T-cell–myeloid hubs may form within tumor tissue without B-cell enrichment, follicular organization, or germinal-center features. In CRC, an intratumoral immune hub containing IFN-responsive myeloid cells and CXCL13^+^, PD-1^+^ T cells was anatomically and compositionally distinct from conventional TLSs (7).

An immune-inflamed ecotype is therefore not adequately defined by CD8^+^ T-cell abundance alone. Its assignment is strengthened by evidence of intranest cytotoxic lymphocytes, effective tumor–immune proximity, granzyme-associated effector activity, IFN-γ-responsive chemokine signaling and, where detectable, supportive NK-cell, cDC1, stem-like T-cell or lymphoid-organizing niches. At the same time, PD-L1, IDO1 and other inhibitory molecules may be induced locally as adaptive counter-regulation (7, 21). Their presence does not negate an inflamed state, but instead demonstrates that immune activation and suppression can coexist within the same regional circuit.

Immune-excluded regions. Immune-excluded regions contain recruited lymphocytes that fail to penetrate malignant-cell territories. Phenotype can occur at several anatomical scales. Margin-restricted tumors accumulate CD8^+^ T cells around the tumor perimeter but contain few lymphocytes in the central parenchyma. Stroma-restricted tumors may contain abundant CD8^+^ cells within the tumor mass, but retain them in connective-tissue compartments outside epithelial nests (21). Multiplexed imaging in triple-negative breast cancer similarly distinguished mixed tumors, in which immune and malignant cells were intermingled, from compartmentalized tumors showing clear spatial segregation despite comparable overall immune infiltration (2).

Cancer-associated fibroblasts (CAFs) and the extracellular matrix can establish both molecular and physical barriers to lymphocyte entry. In metastatic urothelial carcinoma, immune-excluded tumors contained CD8^+^ T cells predominantly within fibroblast- and collagen-rich peritumoral stroma, and this architecture was associated with a TGF-β-responsive fibroblast program and resistance to PD-L1 blockade (19). In viable human lung-tumor slices, T cells migrated preferentially through loose collagen- and fibronectin-rich regions, whereas dense and aligned matrix structures restricted displacement and guided lymphocytes parallel to tumor-cell boundaries, thereby reducing entry into malignant nests (27). More recent spatial profiling of human lung tumors has further linked defined CAF positioning and matrix-remodeling programs to T-cell exclusion (28).

Dysfunctional tumor vasculature may create an additional barrier upstream of the fibroblastic and extracellular-matrix compartments. Angiogenic signaling can induce tumor endothelial-cell anergy, characterized by reduced responsiveness to inflammatory cues, diminished expression of leukocyte-adhesion molecules and impaired leukocyte–endothelium interactions (29). Additional endothelial programs can selectively limit T-cell homing: endothelin B receptor expression in human ovarian tumor endothelium was associated with the absence of tumor-infiltrating lymphocytes, whereas its inhibition restored ICAM1-dependent T-cell adhesion and tumor homing in experimental systems (30). Tumor endothelial cells can also express Fas ligand, which preferentially eliminates effector CD8^+^ T cells while sparing regulatory T cells (Tregs) owing to their higher resistance to Fas-mediated death (31). Immune exclusion may therefore begin at the vascular interface before infiltrating cells encounter fibroblastic or matrix-associated barriers.

Macrophages can reinforce exclusion after lymphocytes have entered the stromal compartment. Dynamic imaging of viable human lung tumors revealed prolonged contacts between stromal macrophages and CD8^+^ T cells that were associated with reduced T-cell motility. In mouse tumor models, CSF-1R inhibition and macrophage depletion increased CD8^+^ T-cell migration, penetration into malignant-cell nests and direct contact with tumor cells (32). Stromal accumulation should therefore not be interpreted as successful tumor infiltration, because lymphocytes may remain physically retained and functionally separated from their targets.

The defining characteristic of an immune-excluded ecotype is therefore not low lymphocyte abundance but spatially interrupted access to malignant cells. Its assessment should distinguish immune cells located at the invasive margin, in peritumoral or intratumoral stroma and within malignant-cell nests. We propose that spatial analyses should quantify tumor–immune distances, boundary crossing, tumor-to-stroma immune-cell ratios, matrix density and orientation, vascular accessibility and the proportion of cytotoxic lymphocytes achieving direct contact with malignant cells (33, 34).

Immune-desert regions. Immune-desert regions show limited immune-cell recruitment in both peripheral and intratumoral compartments. In the spatial classification of triple-negative breast cancer, tumors assigned to the immune-desert category had low CD8^+^ T-cell density at both the tumor core and margin, whereas excluded tumors retained lymphocytes outside malignant-cell territories. Immune-desert tumors were also linked to fibrotic programs, B7-H4 expression, and unfavorable clinical features, although no single feature was shared by all tumors in this category (21).

Defective antigen presentation may contribute to immune paucity. Reduced or absent HLA class I expression was detected in subsets of immune-desert, margin-restricted and stroma-restricted tumors, but not in fully inflamed lesions. Loss of antigen presentation may weaken cytotoxic recognition and prevent establishment of the inflammatory feedback required to sustain lymphocyte recruitment. Because HLA-I loss occurred in only a subset of non-inflamed tumors, however, it should be considered one possible mechanism rather than a defining feature of every immune desert (21, 35).

Weak chemokine signaling provides another route to limited immune recruitment. Regions lacking coordinated CXCL9, CXCL10, CXCL11 and CCL5 expression may fail to establish the gradients required to attract and retain CXCR3^+^ or CCR5^+^ effector populations. A desert designation should therefore incorporate not only low immune-cell density but also regional evidence of deficient recruitment signals, while recognizing that the relevant chemokine programs may vary across tumor types (36–38).

Poor vascularization, disorganized perfusion and endothelial dysfunction may further reduce leukocyte delivery. Angiogenesis-associated endothelial anergy can impair endothelial responsiveness, adhesion-molecule expression and leukocyte extravasation (29). Severe hypoxia can restrict T-cell entry and impose metabolic and adenosinergic constraints on immune-cell function. Nevertheless, hypoxia, necrosis and poor perfusion are not synonymous with an immune desert. Hypoxic or perinecrotic regions may instead contain abundant suppressive myeloid populations or inflammatory neutrophil–monocyte communities (7, 39). Experimental alleviation of tumor hypoxia has increased T-cell infiltration and enhanced T-cell-dependent antitumor activity in selected models, but this does not establish hypoxia as a universal cause or marker of immune absence (40, 41).

A rigorous immune-desert designation therefore requires direct regional evidence of limited immune recruitment. Low vascular density, hypoxia, necrosis, impaired antigen presentation and weak chemokine expression may contribute to this state, but none is individually sufficient to define it. This distinction prevents anatomically hostile but immune-infiltrated territories from being incorrectly classified as deserts when they may instead represent excluded or suppressed ecotypes.

Immune-suppressed regions. We define immune-suppressed regions as immune-infiltrated territories in which antitumor activity is disproportionately limited. They differ from immune deserts because recruitment has occurred and from immune-excluded regions because at least some lymphocytes access malignant-cell territories. Suppression may arise from terminal T-cell dysfunction, regulatory lymphocytes, macrophage- or neutrophil-dominated programs, validated myeloid-derived suppressor cell populations and spatially localized checkpoint or inhibitory circuits.

T-cell-rich regions provide direct evidence that infiltration alone is insufficient. Distinct CD8^+^ T-cell states can coexist within immune-infiltrated tumors, ranging from progenitor-like and effector-like populations to terminally dysfunctional PD-1^hi cells expressing ENTPD1/CD39, TOX2, CTLA4 and HAVCR2. The spatial association of progenitor-like CD8^+^ T cells with CXCL13^+^ helper T cells and LAMP3^+^ mature regulatory dendritic cells further indicates that T-cell fate depends on the cellular niches in which these populations are embedded, rather than solely on their abundance (24).

Regulatory T cells and macrophages can form spatially linked suppressive compartments. At the colorectal invasive front, Treg abundance within a macrophage-enriched neighborhood was negatively correlated with proliferating CD8^+^ T cells in an adjacent T-cell-enriched compartment in diffuse inflammatory tumors. Greater spatial coupling between T-cell- and macrophage-enriched neighborhoods was also accompanied by altered inferred communication with the tumor boundary (4). Regional immunosuppression may therefore depend on interactions between neighboring cellular communities rather than on a single inhibitory population.

Neutrophil-rich inflammation may also be spatially distinct from productive cytotoxic immunity. In CRC, a luminal inflammatory hub comprises coordinated malignant, myeloid and fibroblast programs involving neutrophils, monocytes, IL1B, CXCL1 and CXCR1/2-associated signaling. This hub was concentrated at the interface with the colonic lumen, particularly near necrotic territories, and was anatomically distinct from intratumoral CXCL9–CXCL11-associated T-cell foci (7). A region can therefore be highly inflammatory while being dominated by tissue-damage, wound-healing and potentially tumor-promoting myeloid programs rather than effective tumor-cell killing.

Myeloid-derived suppressor cells should be considered only when their identity is supported by appropriate phenotypic criteria and evidence of suppressive activity. In human tumors, candidate myeloid-derived suppressor cells (MDSCs) overlap phenotypically with conventional monocytes and neutrophils, and suppressive function cannot be inferred reliably from a limited marker panel (42). Their contribution should therefore be distinguished from broader myeloid infiltration and interpreted according to established characterization standards. Where validated, monocytic or polymorphonuclear MDSCs may contribute to regional suppression through arginine depletion, reactive oxygen and nitrogen species, inhibitory cytokines, lipid mediators or disruption of antigen-specific T-cell responses (43).

Checkpoint and inhibitory-mediator expression must likewise be assigned to its cellular and anatomical source. In mixed triple-negative breast tumors, PD-L1 and IDO were expressed predominantly by malignant cells, whereas PD-1 was enriched on CD8^+^ T cells. In compartmentalized tumors, PD-L1 and IDO were preferentially associated with immune cells, whereas PD-1 was more frequently detected on CD4^+^ T cells (2). Tumors with superficially similar overall expression of PD-1, PD-L1 or IDO may therefore contain fundamentally different regulatory interfaces.

An immune-desert designation requires regional evidence of limited immune recruitment. Low vascular density, hypoxia, necrosis, impaired antigen presentation, and weak chemokine expression may accompany immune-desert regions, but none is sufficient for classification on its own. Regions that remain immune-infiltrated despite adverse anatomical or physiological conditions are better classified according to evidence of immune exclusion or local suppression rather than immune desert.

Intratumoral coexistence of immune states. The four ecotypes should not be interpreted as mutually exclusive labels for entire tumors. Regional discordance between the tumor center and invasive margin was already evident in colorectal immune-contexture studies, in which immune-cell densities could differ between these anatomical compartments within individual lesions (5). Multiplexed imaging subsequently demonstrated that tumors with similar conventional lymphocyte scores could display markedly different degrees of tumor–immune mixing, ranging from mixed to compartmentalized architectures, together with distinct cellular sources of PD-1, PD-L1 and IDO (2).

Distinct functional neighborhoods may also coexist within the same lesion. Colorectal tumors contained a luminal, neutrophil- and myeloid-rich inflammatory hub together with intratumoral foci comprising IFN-γ-responsive malignant and myeloid cells and CXCR3-ligand-associated T cells (7). In melanoma, mature secondary-follicle-like TLSs containing B cells, T cells, follicular dendritic cells and germinal-center-associated features can be distinguished from less-organized lymphoid aggregates that lack equivalent follicular architecture (26, 44). Thus, even within a tumor classified globally as inflamed, one territory may support cytotoxic lymphocyte recruitment, another may retain T cells in fibrotic stroma, another may be dominated by suppressive myeloid programs and another may contain few immune cells.

The prognostic or functional importance of an immune population may likewise depend on the neighborhood in which it is embedded. In CRC with diffuse inflammatory infiltration, the abundance of PD-1^+^ CD4^+^ T cells within a granulocyte-enriched cellular neighborhood was associated with overall survival, whereas their overall tissue frequency and the overall abundance of that neighborhood were not (4). This illustrates why global cell counts can obscure biologically meaningful local relationships and why phenotypically similar cells may have different clinical associations according to their spatial context.

In non-small cell lung cancer (NSCLC), 41-plex single-cell spatial proteomics identified ten recurrent cellular neighborhoods corresponding to the tumor core, a macrophage-enriched niche, a B-cell niche, a stromal–fibrotic domain, a plasma-cell cluster, a neutrophil-rich zone, a tumor–immune interface, a T-cell compartment, a pan-immune activation zone, and a vascular niche. Clinical associations reflected multicellular architecture: tumor-core, fibrotic, and neutrophil-rich neighborhoods were linked to unfavorable outcomes, whereas B-cell, plasma-cell, T-cell, and tumor–immune-interface neighborhoods were associated with more favorable outcomes (45). Spatial tumor–immune–stromal configurations therefore provided information beyond overall cell-type composition, supporting regional immune states as properties of multicellular tissue organization rather than isolated immune populations.

The proposed ecotypes should not be interpreted as sequential stages or as states that necessarily coexist within every tumor. Most spatial studies are cross-sectional, and regional coexistence alone cannot establish whether inflamed territories progress toward excluded, suppressed, or desert states (2, 4, 7). Longitudinal multiregional sampling will be required to reconstruct temporal transitions and determine whether regional ecotypes remain stable, reverse, or shift between states over time.

Spatial immune assessment should therefore report immune-cell density and phenotype separately in the invasive margin, peritumoral stroma, intratumoral stroma, tumor center and malignant-cell nests. It should also consider tumor–immune distances, direct cell–cell contacts, cDC1 and NK-cell support, macrophage, neutrophil, MDSC and Treg organization, regional IFN-γ and chemokine activity, antigen-presentation competence, vascular accessibility and the presence of stem-like T-cell niches or TLSs. Where sufficiently resolved data are available, assessment should additionally quantify cellular-neighborhood composition, inter-neighborhood adjacency, spatial segregation, stromal barrier architecture and higher-order multicellular motifs, because these properties may distinguish immune-active from immune-restricted regions even when overall cell-type abundances are similar (2, 4, 45). A global “hot” or “cold” designation may remain useful as a descriptive summary, but it should not be treated as a mechanistic representation of the tumor immune microenvironment. The biologically relevant unit is the spatial immune ecotype: a locally organized cellular community whose composition, accessibility, functional competence and interaction topology determine whether immune activity is absent, spatially excluded, functionally suppressed or productively engaged (46).

## Spatial biogeography and cellular compartmentalization of the intratumoral microbiome

Available spatial studies indicate that the intratumoral microbiome is frequently non-uniform, with microbial signals concentrated in discrete microniches and absent or below detection in adjacent territories. In CRC and OSCC, RNA *in situ* hybridization and spatial transcriptomics have revealed densely colonized regions beside microbe-negative areas within the same tissue section. Spatial localization adds information on anatomical origin, host-cell association, and biological context that is not captured by tumor-wide microbial measurements alone (12).

Tumor microbial biogeography can be conceptualized as the intersection of four processes: route of entry, anatomical filtering, cellular sequestration and local ecological selection. Together, these processes may determine whether microbial material remains associated with a mucosal surface, enters malignant or immune cells, occupies extracellular spaces, persists during tumor dissemination or becomes enriched in particular physiological territories (8, 15). Microbial DNA, RNA, peptides, metabolites, structural products, intact cells and cultivable organisms represent distinct forms of evidence and should not be treated as biologically equivalent (8, 11).

Potential routes of microbial entry. The routes by which microorganisms reach tumor tissue are likely to depend on the anatomy of the organ, proximity to colonized surfaces, epithelial and vascular integrity, and disease stage. No single mechanism can be assumed to operate across all cancers. Direct extension from adjacent mucosal surfaces is most strongly supported in tumors arising at microbe-rich epithelial interfaces. In CRC, fluorescence *in situ* hybridization and scanning electron microscopy identified invasive polymicrobial biofilms penetrating the mucus layer and contacting the epithelium. Biofilm-positive tumors were commonly accompanied by related biofilms on distant tumor-free mucosa, supporting a regional mucosal field rather than microbial organization confined to the malignant lesion (13).

Barrier disruption does not necessarily produce a continuous microbial front. In familial adenomatous polyposis, bacterial mucus invasion occurred in short, discontinuous patches rather than the extensive surface biofilms observed in many sporadic proximal CRC (47).

An anatomically plausible route has been proposed for pancreatic tumors because the pancreatic duct communicates with the duodenum. Bacterial DNA profiles in pancreatic and duodenal tissues from the same individuals showed substantial similarity, which is compatible with microbial movement between these anatomical sites. These findings remain indirect and do not establish retrograde ductal migration as the source of microbial material in every pancreatic tumor (48).

Hematogenous dissemination provides a plausible route for tumors without direct continuity with heavily colonized mucosa and for microbial transfer to distant organs. In CRC models, disruption of the gut vascular barrier permitted microorganisms or microbial material to enter the circulation and reach the liver. In human CRC, vascular-barrier alteration was associated with greater bacterial detection within liver metastases than in paired non-malignant hepatic parenchyma. The experimental evidence supports blood-borne dissemination in this context, whereas the human findings establish an association but cannot independently determine its direction or timing (49).

Microorganisms may also persist alongside disseminating tumor cells. In a murine breast-cancer model, bacterial signals were relatively uncommon in individual circulating tumor cells but were enriched in circulating tumor-cell clusters and subsequent metastatic foci. Bacteria carried by circulating tumor cells promoted resistance to fluid shear stress and metastatic colonization (10).

Human CRC provides complementary evidence of microbial persistence across metastatic progression. Cultivable bacterial strains recovered from matched primary tumors and liver metastases collected months or years apart showed greater than 99.9% average nucleotide identity. This similarity is compatible with transfer or stable persistence during dissemination but does not establish the specific transport mechanism (15).

Evidence for direct lymphatic dissemination remains limited. Bacterial antigen staining has been detected in primary breast tumors and lymph-node metastases, but these observations do not establish strain continuity or determine whether microbial material reached the lymph node inside metastatic cells, as free organisms, within leukocytes or from the pre-existing lymph-node environment (50).

Immune cells may contribute to microbial trafficking, although direct transport into tumors has not been demonstrated. Microbial RNA and structural material have been detected in macrophages and other CD45^+^ cells, and single-cell analyses have assigned bacterial transcripts to monocyte-derived macrophage populations (8, 12).

Anatomical compartments. The luminal surface and adjacent mucosa constitute major microbial compartments in gastrointestinal tumors. In sporadic CRC, tumors harboring biofilms were generally accompanied by biofilms on macroscopically normal mucosa distant from the lesion. Biofilm invasion extended deeper within tumor tissue, whereas bacterial density was comparable between tumors and paired biofilm-bearing mucosa. The distribution is consistent with a regional mucosal field rather than microbial organization restricted to the malignant mass (13).

Adjacent tissue should not automatically be treated as a sterile reference. Across several cancer types, tumor-adjacent tissues contained detectable microbial signals, although the relationship between tumor, adjacent tissue and non-cancer tissue varied by organ. In breast cancer, tumor-adjacent tissue showed bacterial characteristics intermediate between healthy breast tissue and tumor tissue (8). Fungal communities likewise showed substantial similarity between paired tumor and adjacent-tissue samples, although these patterns were cancer-type dependent (51). Such continuity may reflect a shared regional ecosystem, field alteration or secondary influence from the tumor, but does not establish the direction of microbial transfer.

Within the tumor mass, microorganisms can occur in both the core and invasive margin, although their distribution is often highly uneven. In primary CRCs, invasive bacterial signals were predominantly focal and located in isolated malignant-appearing cells or small cellular groups near luminal and ulcerated regions. Less frequent signals were observed in glandular structures within the tumor center and invasive margin. In liver metastases, bacteria were found mainly in cells morphologically consistent with metastatic cancer cells, with occasional stromal localization and no detection in adjacent residual hepatic parenchyma (15).

The tumor–stroma interface should consequently be analyzed separately from malignant-cell nests and surrounding non-malignant tissue. Whole-tissue sequencing cannot determine whether a signal originates from malignant cells, fibroblast-rich stroma, extracellular matrix-associated spaces or immune cells crossing the epithelial–stromal boundary. Available studies document occasional stromal microorganisms, but do not yet establish a universal pattern of enrichment at this interface (8, 15).

Hypoxic territories may favor microorganisms adapted to low-oxygen conditions. In a cohort of 4,160 metastatic biopsies, anaerobic bacterial profiles were enriched in tumors with stronger hypoxia-associated signatures and reduced oxygen-response programs. An association with hypoxia was also examined in head-and-neck tumors using HIF-1α staining. Tumor-level correlations between microbial profiles and hypoxia, however, do not establish colocalization of individual microorganisms with hypoxic cells within the same histological microniches (14).

Spatial profiling has provided more direct evidence for associations with particular vascular and proliferative contexts. In oral and colorectal tumors, bacteria-positive epithelial microniches showed reduced smooth-muscle-actin-associated vascular features and lower Ki-67 and p90 RSK expression relative to contiguous bacteria-negative regions (12). These observations support an association between bacterial localization and less vascularized, lower-proliferation territories, while leaving unresolved whether the tissue state preceded microbial localization or emerged after it.

A generalized perivascular microbial reservoir has not been established. Imaging mass cytometry combined with bacterial FISH in colorectal liver metastases showed CD31^+^ vascular networks surrounding metastatic lesions, while bacterial signals were detected within SOX9^+^ malignant-cell regions. The vascular architecture therefore provides anatomical context for bacteria-positive lesions but does not demonstrate preferential microbial localization adjacent to blood vessels. Direct measurements of vessel-to-microbe distance are needed before perivascular localization can be regarded as a recurrent spatial feature (49).

Necrotic regions are similarly plausible microbial reservoirs because they combine low oxygen availability, altered nutrients and tissue disorganization. Nevertheless, the available studies do not provide sufficient direct histological evidence that microorganisms are consistently enriched within necrotic or perinecrotic tissue. Necrosis, viable hypoxia and poor perfusion should therefore be annotated as separate spatial variables rather than grouped together as established bacteria-rich compartments.

Metastatic lesions constitute distinct ecological sites rather than passive copies of primary tumors. Analysis of more than 4,160 metastatic biopsies showed that microbial community composition was strongly influenced by the anatomical location of the metastasis, in some analyses more than by the tissue of origin of the primary cancer. These findings support organ-specific ecological selection within metastatic sites (14).

Cellular compartments. Malignant cells are among the most strongly supported cellular compartments containing intracellular microbial signals. Across multiple human cancers, bacterial 16S rRNA and structural signals were detected mainly in cancer and immune cells. Within malignant cells, bacterial RNA was predominantly cytoplasmic, and correlative light and electron microscopy confirmed intracellular bacterial structures in human breast tumors, often close to the nuclear membrane but not within the nucleus (8).

In OSCC, bacterial transcripts were preferentially assigned to malignant epithelial cells and monocyte-derived macrophages. Microbial transcript abundance also differed among epithelial subpopulations, demonstrating that cellular association is heterogeneous even within the malignant compartment (12).

Macrophages frequently contain microbial material, but the nature of that material must be specified. In pan-cancer analyses, macrophages commonly displayed strong bacterial cell-wall staining but relatively infrequent 16S rRNA signals (8). Because structural components may persist after phagocytosis and degradation, macrophage positivity for lipopolysaccharide or lipoteichoic acid cannot independently establish viable intracellular microorganisms. It may instead reflect previous exposure, engulfment or prolonged retention of microbial products.

Other immune populations can also contain microbial signals. CD45^+^CD68^-^ cells showed cytoplasmic bacterial RNA in selected tumors (8), and fungal imaging revealed cancer-type-dependent localization of fungal signals in malignant or immune cells (51). Broad immune markers, however, do not distinguish neutrophils, dendritic cells, lymphocytes and other leukocyte populations. Specific cellular compartments therefore require multiplexed phenotyping, single-cell analysis or direct cell-resolved imaging rather than CD45 staining alone.

Neutrophils were enriched around bacteria-positive microniches in patient tumors and experimental models, but direct intracellular residence was not demonstrated with the same resolution as in malignant cells. In experimental spheroids, neutrophils accumulated and formed clusters around bacteria-containing cancer-cell structures, paralleling their enrichment in bacteria-positive regions of human tumors (12). Neutrophil accumulation should therefore be interpreted as spatial association unless intact intracellular microorganisms are demonstrated by cell-specific imaging.

Extracellular microbial organization is highly context-dependent. In mucosal colorectal tumors, polymicrobial biofilms can occupy the mucus layer and directly contact epithelial cells (13). By contrast, electron microscopy in a murine breast-cancer model located approximately 97% of detected bacterial structures within the cytosol rather than extracellular spaces (10). Intracellular predominance in this experimental model should not be generalized to human cancers arising at colonized mucosal surfaces.

The term biofilm should be restricted to microbial communities supported by direct structural evidence. In colorectal tissue, biofilms were defined as dense bacterial invasion of the mucus layer extending for at least 200 μm along the epithelial surface. Their organization was demonstrated using mucus-preserving fixation, pan-bacterial and group-specific FISH, confocal microscopy and scanning electron microscopy showing mixed microbial morphologies and direct epithelial contact (13). Isolated intracellular puncta, spatial-transcriptomic hotspots or clusters of microbial sequencing reads should not be designated as biofilms without comparable histological evidence.

Regional Microbial–Host Organization and Intratumoral Heterogeneity. Regional heterogeneity is a defining feature of the intratumoral microbiome in tumors examined with spatially resolved approaches. Analysis of separated pieces from individual colorectal tumors showed relatively stable microbial composition in only a minority of cases, while RNA *in situ* hybridization demonstrated densely colonized microniches adjacent to regions with little or no detectable bacterial signal. Direct comparison of bacteria-positive and bacteria-negative regions in OSCC and CRC further showed greater CD11b^+^ and CD66b^+^ myeloid-cell representation, increased ARG1-associated signals, fewer CD4^+^ and CD8^+^ T cells, reduced smooth-muscle-actin-associated vascular features, and lower Ki-67 and p90 RSK expression in bacteria-positive territories. These regional differences establish a spatial association between bacterial localization and a distinct local tissue configuration, while the direction of that relationship remains unresolved (12).

Regional co-occurrence should not be equated with direct cell-to-microbe contact. Available evidence supports myeloid enrichment around bacteria-containing territories and microbial assignment to malignant epithelial cells and monocyte-derived macrophages, while occasional stromal signals have also been reported without evidence of recurrent enrichment at the tumor–stroma interface. Spatial transcriptomics further showed that bacterial transcripts were restricted to subsets of tissue locations, occurring in 28% and 46% of capture spots in the examined oral and colorectal tumors, respectively. Because individual spots may contain several cells and extracellular material, regional localization and cellular compartmentalization remain complementary but non-equivalent forms of evidence (8, 12, 14).

Cell-resolved analyses provide additional information on the host compartments containing microbial material. Across multiple tumor types, microbial signals have been detected within malignant and immune cells, and OSCC analyses preferentially assigned bacterial transcripts to malignant epithelial cells and monocyte-derived macrophages. Microbial transcript abundance also differed among epithelial subpopulations. Cell-resolved measurements identify the host population associated with a microbial signal, whereas reconstruction of its relationship with neighboring lymphocytes, stromal cells, or vessels requires preservation of the original tissue architecture (8, 12).

The physical extent of microbial distribution also varies within colorectal tumors. Invasive bacterial signals in primary CRC were frequently restricted to isolated malignant-appearing cells or small cellular groups near luminal and ulcerated regions, with less frequent detection in glandular structures within the tumor center and invasive margin. In contrast, many sporadic proximal colorectal tumors contained broad polymicrobial surface biofilms, whereas familial adenomatous polyposis showed shorter, discontinuous patches of mucosal bacterial invasion. Total microbial burden alone therefore cannot distinguish focal intracellular or multicellular localization from extensive mucosal colonization (13, 15, 47).

Microbial localization was also associated with regional proliferative state. Within the same tumors, bacteria-positive microniches contained fewer Ki-67^+^ cells and lower proliferative signaling than adjacent bacteria-negative regions. The finding links bacterial localization with reduced proliferative activity at the regional level, although the direction of the relationship and its generalizability across highly proliferative tumor regions remain unresolved (12).

Spatial heterogeneity extends across disease sites. Highly similar viable bacterial strains have been recovered from selected matched colorectal primary tumors and liver metastases, whereas primary–metastatic pairs with low or undetectable primary-tumor burden showed less community concordance. Experimental breast-cancer models likewise demonstrated persistence of selected microbial populations during early dissemination followed by community changes during metastatic growth. Primary and metastatic sites may therefore retain selected microbial populations while undergoing additional site-specific ecological filtering (10, 14, 15).

A single biopsy may consequently provide an incomplete representation of microbial composition and microbial–host spatial organization. Multiregional sampling should distinguish, where anatomically relevant, the luminal surface, adjacent mucosa, tumor center, invasive margin, malignant-cell nests, tumor–stroma interface, physiologically distinct regions, and matched metastatic sites, while recording whether microbial signals are focal or diffuse and intracellular or extracellular. Tumor-level associations can therefore serve to identify candidate taxa or host processes for spatial investigation, whereas direct regional localization is required to determine whether microbial and host features share the same tumor microniche (12, 14, 15).

Microbial viability and activity. Microbial presence, localization, structural integrity, viability and functional activity are complementary dimensions of evidence rather than successive steps in a simple hierarchy (8, 52, 53). Microbial DNA establishes the presence of genetic material. Amplicon sequencing, whole-genome sequencing and metagenomic assembly can identify microbial taxa or genomes, but cannot distinguish viable organisms from dead cells, extracellular DNA or contamination. This limitation is especially consequential in low-biomass tumors, for which extraction controls, no-template controls, paraffin controls and batch-aware decontamination are essential (53, 54).

Microbial RNA is more compatible with transcriptionally preserved or recently viable organisms than DNA alone and can be localized using rRNA FISH or spatial transcriptomics. RNA detection does not, however, independently demonstrate replication or cultivability because ribosomal RNA may persist transiently after viability is lost. Its interpretation is strongest when combined with intact morphology, cell-resolved localization or successful culture (52, 55).

Microbial peptides provide evidence of microbial protein material, whereas HLA-bound microbial peptides demonstrate its uptake and processing by host cells. Neither observation independently establishes the continued presence, localization or current activity of an intact microorganism. Peptide evidence should therefore be interpreted separately from microbial-cell detection (11).

Microbial metabolites may indicate microbial biochemical capacity or activity, but source attribution is particularly difficult. Many metabolites can be generated by both host and microbial pathways or can diffuse into tumors from distant anatomical compartments. Without spatial colocalization, isotope tracing or organism-specific enzymatic evidence, a metabolite cannot be assumed to have been produced by microorganisms residing in the same tumor region (56).

Structurally intact microbial cells require direct visualization. Correlative light and electron microscopy has confirmed intracellular bacterial structures in human breast tumors, while electron microscopy in experimental models has identified cytosolic organisms with recognizable bacterial morphology. Imaging establishes anatomical position and structural preservation but cannot determine the viability of every visualized organism (8, 10).

Cultivable microorganisms provide direct evidence that at least a subset of tumor-associated organisms was viable at the time of tissue collection and capable of growth under the tested conditions. Broad culturomics recovered numerous bacterial colonies from fresh human breast tumors with minimal growth in parallel negative controls. Viable microorganisms were also cultured from paired colorectal primary tumors and liver metastases and recovered through serial patient-derived xenograft passages. Culture is strong evidence of viability, but underestimates organisms requiring untested nutrients, host-cell dependence or specialized environmental conditions (8, 57).

Functionally active microorganisms require direct evidence of ongoing transcription, translation, metabolic activity, replication or a measurable microbial function. Persistence across time, metastatic dissemination or serial passage supports stable residence and, when accompanied by successful culture, viability; it does not independently demonstrate *in situ* functional activity. The strongest assignment of activity therefore combines orthogonal evidence, such as microbial transcription, intact morphology, signs of cellular division, cultivation or direct measurement of organism-specific biochemical function. Such evidence has been obtained in selected experimental and clinical settings, but cannot be extrapolated to every microbial sequence detected in tumor tissue (56). Structural products such as lipopolysaccharide, lipoteichoic acid and β-glucan require separate interpretation. They may remain detectable after microorganisms have been killed or processed and may accumulate in phagocytic cells. Their presence establishes exposure to microbial material, not necessarily an intact or viable tumor-resident community (51).

The relevant unit of analysis is therefore a spatially and evidentially defined microbial niche: an anatomical region containing a specified form of microbial evidence, associated with an identifiable host or extracellular compartment and accompanied by an explicit level of confidence regarding microbial integrity, viability and activity. Tumor-wide taxonomic abundance alone cannot reveal whether a signal originates from a mucosal biofilm, an intracellular malignant-cell reservoir, a phagocytic immune cell, extracellular microbial material or a metastatic microbial population. A rigorous understanding of the intratumoral microbiome requires the joint resolution of three coordinates: where the microbial signal is located, which compartment contains it and what biological state it represents (11, 56).

## Local microenvironmental conditions shaping intratumoral microbial localization

Solid tumors contain uneven oxygenation, perfusion, pH, nutrient delivery, and mechanical pressure. Tumor expansion, stromal deposition, and elevated interstitial pressure can compress blood vessels and reduce perfusion, generating hypoxic regions that may favor facultative and obligate anaerobes. In metastatic tumors, anaerobic bacterial abundance correlated positively with hypoxia-related transcriptional signatures and negatively with oxygen-response programs. Head and neck tumors with stronger HIF-1α staining showed a comparable enrichment of anaerobic taxa. The association is consistent with hypoxia acting as a selective condition, although available data do not establish whether hypoxia precedes microbial colonization or is partly reinforced by microbial activity (14, 58).

Microbial access and persistence may also be influenced by tissue damage and vascular architecture. Loss of epithelial and mucus barriers can permit entry from adjacent mucosal communities, whereas permeable tumor vessels may enable hematogenous dissemination. Necrotic areas contain cellular debris and locally released nutrients that can support microbial survival despite poor vascular delivery. Intracellular localization may provide protection from immune clearance and access to host-cell nutrients (59, 60). The poorly vascularized and lower-proliferation microniches described above are compatible with local ecological selection, although available data do not establish whether the tissue condition preceded microbial localization (12).

Several additional physicochemical factors may contribute to microbial niche selection, although direct spatial evidence remains limited. Some anaerobic bacteria produce lactate during pyruvate fermentation, but microbial lactate production has not been quantified in spatially defined human tumor microniches. Biofilms have been detected in colorectal tumor tissue, yet internal oxygen gradients, metabolically inactive bacterial populations, and increased stress tolerance have not been demonstrated within tumor-associated biofilms. A role for local iron availability in shaping intratumoral microbial localization also remains untested. Acidity, biofilm-associated gradients, iron availability, and local nutrient conditions should therefore be considered candidate selective pressures rather than established determinants of intratumoral microbial distribution (12, 61, 62).

Malignant cells, activated lymphocytes, myeloid cells, and microorganisms may depend on overlapping pools of glucose and amino acids. Current spatial evidence is compatible with altered host arginine metabolism rather than direct microbial competition for nutrients. The previously described bacteria-positive microniches contained increased ARG1 expression, mature CD66b^+^ myeloid cells, and fewer T-cell markers, but regional arginine concentrations and bacterial uptake rates were not measured. These findings therefore cannot determine whether microorganisms induced a nutrient-restrictive condition or preferentially occupied a pre-existing arginine-poor region (12, 63).

Lactate may participate in cross-feeding between hypoxic and better-oxygenated tumor compartments. Fermentative microorganisms and host cells may contribute to the extracellular lactate pool, whereas oxygenated malignant cells can import lactate for oxidative metabolism. The direction and magnitude of this exchange have not been quantified *in vivo*, and the microbial contribution relative to malignant and stromal sources remains unresolved (64, 65).

Cross-feeding may also occur among microorganisms. *F. nucleatum*-positive colorectal tumors show non-random co-colonization with organisms such as *Bacteroides fragilis* and *Prevotella intermedia*, and polymicrobial communities can display greater epithelial invasion than individual species. Non-random co-colonization suggests organized microbial associations, although the exchanged metabolites have not been identified. Demonstration of microbial cross-feeding within tumors will require species-resolved localization combined with metabolic flux analysis rather than co-occurrence alone (66, 67).

The bacteria-positive microniches described above support the possibility that microbial activity and metabolite exposure are spatially compartmentalized rather than uniformly distributed throughout the tumor. Reduced perfusion, hypoxia, acidity, and nutrient limitation could influence the local production, diffusion, and retention of metabolites within these regions. However, metabolite concentrations, microbial pathway activity, and host-cell exposure have not been measured directly within bacteria-positive tumor microniches. Current evidence therefore supports spatially heterogeneous metabolic environments but does not establish that intratumoral microbial metabolism generates the associated immune phenotype (58, 68).

## Regional tumor immunity in microbial niches

### Microbial antigenicity and innate sensing in spatial and cellular context

The immune consequence of an intratumoral microbial signal depends on where that signal is encountered and which host cell processes or senses it. Microbial proteins presented by malignant or antigen-presenting cells can become visible to T cells, whereas microbial structures or nucleic-acid-associated signals can activate innate pathways in malignant or myeloid compartments. Neither process alone defines an immune-active microbial niche. Regional organization additionally requires localization of the microbial signal, the presenting or sensing cell, and the responding immune population within the same tumor territory. Current evidence resolves several components of these interactions but not their complete integration within an intact human tumor niche (11, 69).

Presentation of microbial antigens. Bacteria-derived HLA peptides have been identified directly within human melanoma metastases. Across 17 lesions from nine patients, 248 unique bacterial HLA class I peptides and 35 HLA class II peptides were recovered from microbial species detected in the corresponding tumors. Several HLA-I peptides recurred across metastases from the same patient or between patients, and peptide length, sequence motifs, and synthetic-spectrum validation supported their assignment as authentic HLA ligands. The finding establishes that intratumoral microbial proteins can enter antigen-processing pathways rather than remaining detectable only as microbial DNA or structural material (11).

Microbial HLA peptides occurred in both CD45^-^ and CD45^+^ tumor fractions, indicating antigen processing within malignant and immune-cell compartments. Bacteria-exposed melanoma cells generated HLA-I- and HLA-II-bound microbial peptides, and disruption of B2M or CIITA reduced recovery of the corresponding ligands. Selected HLA-I peptides recovered from tumors also induced IFN-γ secretion and CD69 upregulation in autologous tumor-infiltrating lymphocytes, demonstrating recognition by T cells already present within the lesion. Presentation by malignant cells and presentation by professional antigen-presenting cells therefore represent biologically distinct routes through which microbial material may become immunologically visible. Tissue dissociation, however, removes the regional coordinates needed to determine where peptide-bearing cells and cognate lymphocytes interact *in situ*. Microbial HLA presentation consequently provides strong evidence of intratumoral antigen processing, cellular compartmentalization, and T-cell recognition, but not yet of a spatially organized antigen-specific microniche (11). Compartment-dependent routes of microbial antigen presentation are summarized in **Figure 2A**.

**Figure 2:**
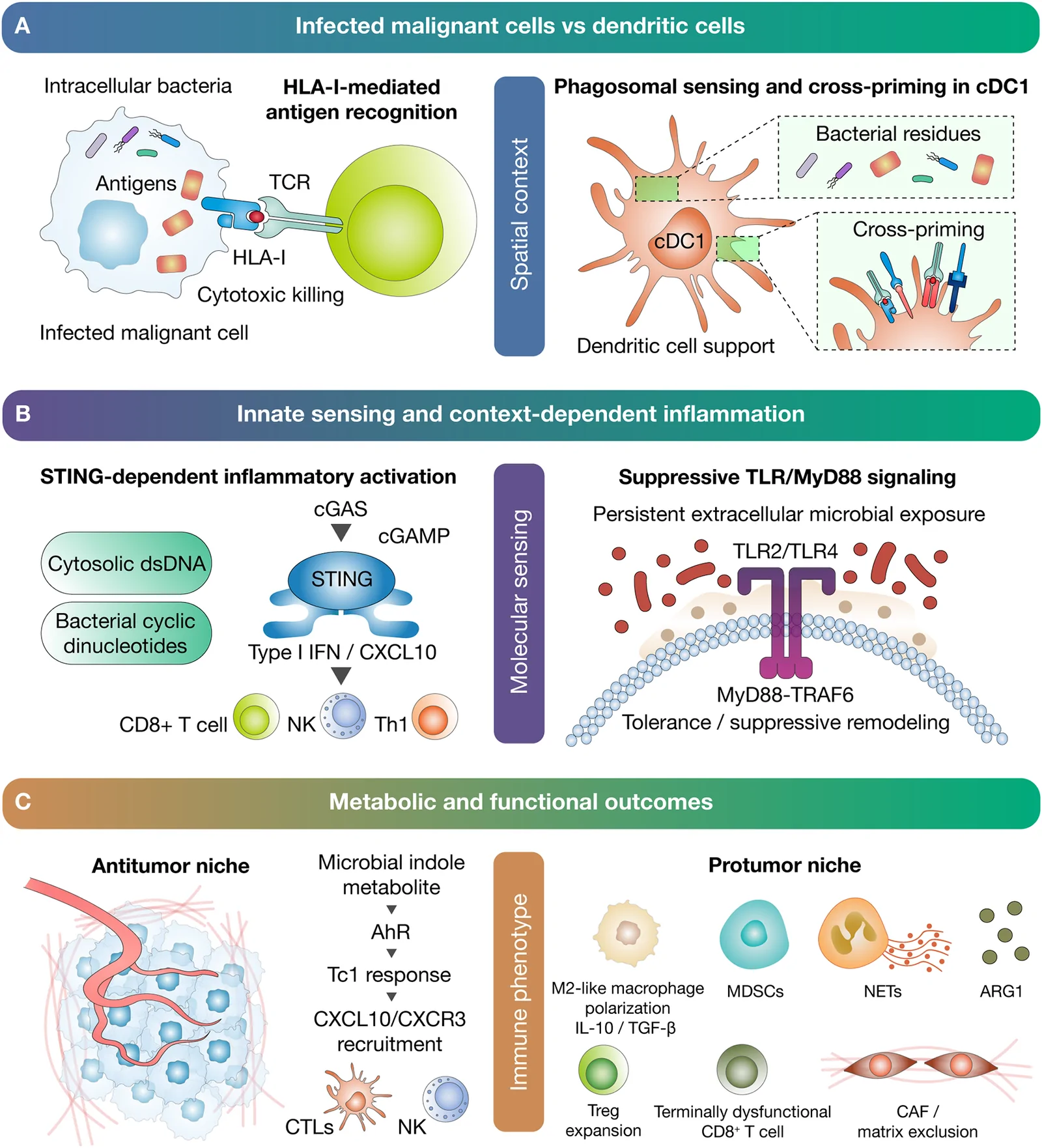
Cellular compartmentalization, molecular sensing, and context-dependent immune consequences of intratumoral microbial signals. Conceptual model illustrating how microbial localization, host-cell identity, molecular sensing, and exposure duration may generate divergent antitumor or protumor immune states. The central vertical labels denote successive levels of interpretation—spatial context, molecular sensing, and immune phenotype—rather than an obligatory temporal or causal sequence. **(A)** Microbial antigen presentation by malignant cells and dendritic cells. On the left, intracellular bacteria or bacteria-derived proteins are processed within infected or bacteria-exposed malignant cells, generating peptides that may be presented on human leukocyte antigen class I (HLA-I) molecules and recognized through the T-cell receptor (TCR) of cognate cytotoxic CD8^+^ T lymphocytes, potentially resulting in malignant-cell killing. On the right, conventional type 1 dendritic cells (cDC1s) are depicted acquiring bacteria or bacteria-containing cellular material and supporting antigen processing, cross-presentation, and cross-priming. HLA peptidomics and functional T-cell assays provide complementary evidence for microbial antigen presentation, although the complete sequence connecting a naturally occurring intratumoral microorganism, a defined cDC1-presented peptide, and its cognate T-cell response has not been spatially resolved in intact human tumors. **(B)** Innate sensing and context-dependent inflammation. Cytosolic double-stranded DNA can activate cyclic GMP–AMP synthase (cGAS), generating cyclic GMP–AMP (cGAMP), which subsequently activates stimulator of interferon genes (STING). Bacterial cyclic dinucleotides, including cyclic di-AMP, can activate STING directly without requiring cGAS. In immune and myeloid-cell contexts, STING-dependent type I interferon and CXCL10 production may support cDC1, NK-cell, and cytotoxic T-cell activity. The schematic emphasizes this immune-supportive branch; however, STING activation is not uniformly antitumoral, because tumor-cell-intrinsic cGAS–STING signaling can instead induce IL-17B-associated protumor neutrophil programs. Persistent exposure to microbial products can engage Toll-like receptor 2 or Toll-like receptor 4 (TLR2/TLR4) and downstream MyD88–TRAF6 signaling. Depending on the responding cell and duration of stimulation, chronic TLR signaling may promote tolerogenic or suppressive myeloid remodeling rather than effective antitumor immunity. **(C)** Metabolic and functional outcomes. The antitumor microniche depicts two complementary but not necessarily causally linked processes: CXCL10–CXCR3-mediated recruitment of effector lymphocytes and microbial indole-3-aldehyde–AhR signaling capable of enhancing IFN-γ-producing type 1 cytotoxic T-cell responses. Their juxtaposition should not be interpreted as evidence of a single continuous pathway within the same tumor region. The protumor microniche summarizes M2-like macrophages and IL-10/TGF-β-associated suppression, MDSC accumulation, ARG1 activity, NET formation, regulatory T-cell expansion, terminal CD8^+^ T-cell dysfunction, and CAF- and extracellular-matrix-associated exclusion. These mechanisms derive from different tumor and experimental contexts and are not presumed to coexist within every microbial microniche. The depicted pathways are non-exclusive, and their consequences depend on microbial identity, burden, viability, localization, host-cell compartment, tumor context, and duration of exposure.

Innate immune sensing. Innate sensing of tumor-associated microorganisms also varies according to the host cell receiving the signal. In pancreatic tumors, microbial exposure promoted suppressive macrophage and monocytic differentiation through TLR–MyD88–TRAF6 signaling and reduced effective T-cell activation. Microbial depletion or interruption of MyD88–TRAF6 signaling diminished the suppressive activity of microbiome-conditioned myeloid cells. TLR engagement therefore did not produce a uniformly stimulatory state; in the pancreatic setting, microbial sensing favored myeloid tolerance and impaired lymphocyte support (70).

STING-dependent sensing can produce a contrasting immune configuration. Bacterial cyclic dinucleotides activated STING in intratumoral monocytes and increased type I interferon production, preserving stimulatory monocyte and dendritic-cell populations while supporting cDC1 accumulation. The same circuit increased CXCL10, CCL2, and IL-18 in myeloid cells and enhanced XCL1 and CCL5 production by intratumoral NK cells. Microbiota depletion, STING deficiency, or IFNAR loss disrupted the immune-supportive configuration and shifted the myeloid compartment toward tumor-promoting macrophage states. Tumor-localized bacterial accumulation also enhanced STING- and IFNAR-dependent dendritic-cell IFN-β production and cross-priming capacity. Intratumoral microbial signals can therefore engage defined innate and antigen-presenting functions, although equivalent receptor-defined microbial–immune interfaces have not yet been mapped within anatomically resolved human tumor regions (69, 71).

Cellular localization can reverse the immune effect of the same bacterial strain. In a breast-tumor model, cytosolic bacteria were associated with double-stranded-DNA accumulation and activation of a tumor-cell-intrinsic cGAS–STING–IL-17B pathway, generating protumor neutrophils that suppressed cytotoxic T-cell activity. When the bacteria remained extracellular, neutrophils instead acquired antitumor activity without equivalent STING engagement. The cytosolic DNA source was not directly resolved, but intracellular and extracellular localization exposed the same microorganism to different host-sensing programs and produced opposing immune phenotypes (72). Cellular address is therefore as relevant as microbial identity when interpreting innate sensing within tumors.

Formation of immune-active niches. Tumor-associated bacterial signals can influence several cellular components required for productive antitumor immunity. Microbial presence has been associated experimentally with type I interferon production, dendritic-cell maturation, NK-cell activity, cDC1 maintenance, cross-priming, and CD8^+^ T-cell recruitment. Loss of tumor-associated bacteria reduced dendritic-cell abundance and CD80 expression together with CD8^+^ T-cell infiltration, whereas STING-dependent microbial sensing promoted monocyte-derived interferon responses and dendritic-cell function (58, 62, 63). NK cells further contributed XCL1, CCL5, and GM-CSF, while interferon-responsive dendritic cells provided reciprocal IL-15-associated support, linking microbial sensing to an NK–DC circuit capable of sustaining an immune-permissive microenvironment (22, 69).

Spatial integration of the reported components remains unresolved. Microbial peptide presentation, TIL recognition, dendritic-cell activation, chemokine induction, NK-cell communication, and CD8^+^ T-cell recruitment have been demonstrated individually or in complementary experimental settings, but cognate interactions among microbial-signal-bearing cells, antigen-presenting cells, and responding lymphocytes have not been visualized together within the same intact human tumor region. Assignment of an immune-active microbial niche would therefore require convergence of a localized microbial signal, an identified sensing or presenting cell, evidence of dendritic-cell activation or antigen presentation, relevant chemokine production, and spatially proximate lymphocytes capable of an appropriate response. Microbial abundance or an interferon-associated transcriptional program alone cannot establish such a niche (11, 69, 71).

Context-dependent consequences. Microbial sensing can generate inflammation without productive antitumor immunity. In pancreatic tumors, TLR-dependent microbial conditioning reduced macrophage antigen-presenting competence and promoted suppressive myeloid differentiation, whereas STING/type I interferon signaling in monocytes and dendritic cells supported immune-cell recruitment and cross-priming. A comparable divergence emerges when bacterial localization changes from extracellular to intracellular compartments: extracellular bacteria can support antitumor neutrophil activity, whereas cancer-cell-intracellular bacteria can engage cGAS–STING–IL-17B signaling and generate suppressive neutrophils. The immune outcome therefore cannot be inferred from pathway activation or microbial presence alone (70, 72, 73).

Tumor-associated microorganisms cannot be classified as intrinsically immunostimulatory or immunosuppressive. Their biological effect depends on microbial localization, the host cell encountering or processing the signal, the pathway engaged, exposure duration, and the immune populations occupying the surrounding region. A microbial signal sensed by an interferon-competent monocyte or dendritic cell may support NK–DC communication and cytotoxic immunity, whereas sustained sensing in suppressive myeloid cells or cytosolic engagement within malignant cells may favor tolerance or protumor inflammation. A spatially defined microbial–immune interaction therefore requires convergence of microbial material, host-cell identity, signaling activity, and neighboring immune composition within the same tumor region rather than inference from tumor-wide abundance or mechanistic observations obtained in isolation (70, 72, 73). Localization-dependent innate-sensing pathways and their divergent immune consequences are summarized in **Figure 2B**.

### Microbiome-associated immune suppression and exclusion

Myeloid Recruitment and Suppressive Niches. The bacteria-positive microniches described above provide the strongest current human spatial evidence linking microbial signals with local myeloid enrichment and reduced T-cell representation. Total microbial burden can influence local immune composition, but quantity alone does not determine the direction of the immune response. Bacterial loads were higher in CRC liver metastases than in non-metastatic colorectal tumors and adjacent liver tissue. In an experimental CRC liver-metastasis model, higher intratumoral *Escherichia coli* abundance increased metastatic burden and was accompanied by accumulation of CD206^+^ macrophages, whereas antibiotic treatment reduced bacterial abundance and the M2-like phenotype. In lung adenocarcinoma, *Aspergillus sydowii* promoted tumor progression despite the low biomass of the intratumoral mycobiome. A biologically active taxon may therefore alter myeloid function without representing a large fraction of the tumor biomass (12, 74, 75).

Microorganisms can recruit several myeloid populations through tumor- and taxon-dependent signals. *Fusobacterium nucleatum* has been associated with increased CD11b^+^ myeloid infiltration, while dysbiotic communities can induce neutrophil-attracting chemokines such as CXCL8. Intratumoral microbial regulation of CCL20 and CXCL8 has also been linked to recruitment of suppressive immune populations and reduced CD8^+^ T-cell function. Cytokines induced by microbial products promoted polymorphonuclear MDSCs (PMN-MDSCs) accumulation during colorectal tumorigenesis, allowing an inflammatory response to acquire a suppressive myeloid composition (76–78).

The immune effect of microbial burden is not consistent across tumor types. High bacterial loads have been associated with fewer lymphoid effector cells and greater densities of macrophages, neutrophils, or MDSCs in several tumor cohorts. In contrast, pancreatic tumors from long-term survivors contained more diverse intratumoral communities together with higher CD4^+^ and CD8^+^ T-cell frequencies. Differences among studies may reflect microbial species and strain, tumor site, treatment status, host compartment, and spatial location. Biomass-dependent and taxon-dependent effects should therefore be evaluated separately rather than inferred from relative abundance alone (79, 80).

Myeloid recruitment may initially represent an antimicrobial response. In *F. nucleatum*-infected CRC spheroids, neutrophils migrated toward infected cells, accumulated within the spheroids, and formed transient clusters. Bacteria-positive regions in human tumors showed similar enrichment of CD66b^+^ and CD11b^+^ cells. Persistent recruitment did not correspond to effective tumor rejection because the same regions contained ARG1 and fewer T-cell markers. Antimicrobial recruitment and antitumor immunity should therefore be considered separate biological outcomes (12, 81).

Available studies support ARG1-associated myeloid niches but do not directly identify microbiota-associated SPP1^+^ or TREM2^+^ macrophage states. ARG1 was elevated within the CD45^+^ immune compartment of bacteria-positive regions in oral and colorectal tumors and coincided with enrichment of CD66b^+^ cells. ARG1 could not therefore be assigned exclusively to macrophages. In lung adenocarcinoma, increased ARG1 activity was attributed mainly to MDSCs. By contrast, CRC liver metastases contained CD206^+^ macrophages with increased ARG1 and MRC1 expression, providing more direct evidence of a microbiota-associated M2-like macrophage state (12, 75).

Macrophage depletion reduced the metastatic burden associated with intratumoral *E. coli* without significantly altering bacterial abundance or intratumoral lactate concentration. CD206 and Foxp3 also declined after depletion. The findings place macrophages downstream of microbial colonization and indicate that their accumulation was not merely a correlate of higher bacterial load (74).

Microbiota-conditioned macrophages can suppress lymphocyte function through soluble mediators. In the colorectal liver metastasis model, lactate-conditioned Kupffer cells increased PD-1, TGF-β, and IL-10 expression in Tregs. The same macrophages reduced CD8^+^ T-cell expansion and decreased IL-2 and IFN-γ expression. In OSCC, dysbiosis involving *F. nucleatum* and *Porphyromonas gingivalis* has been associated with accumulation of M2-like tumor-associated macrophages (TAMs) and production of IL-10, TGF-β, CCL2, and CSF-1. Such mediators may support Tregs and MDSCs while limiting effector T-cell activity (82).

Microbiota-associated macrophages may also contribute to angiogenic and matrix-remodeling programs. However, most supporting evidence derives from the broader tumor-associated macrophage literature rather than spatial studies demonstrating microbial signals, macrophages, and stromal remodeling within the same region. A direct microbiota–macrophage–stroma circuit should therefore not be inferred from macrophage abundance or polarization markers alone (83, 84).

Macrophage-specific defects in antigen presentation remain unresolved because MHC-II expression and co-stimulatory activity were not measured directly. The reported findings instead support impaired CD8^+^ T-cell expansion, reduced effector cytokine production, increased regulatory signaling, and arginase-dependent suppression. Reduced MHC-II expression or defective co-stimulation should not be presented as established features without macrophage-specific measurements (85, 86).

Intratumoral *A. sydowii* promoted MDSC recruitment and expansion in lung adenocarcinoma, with preferential enrichment of PMN-MDSCs in one model. The resulting cells expressed higher *Arg1* and *Nos2* and produced more arginase, ROS, and nitric oxide. MDSCs isolated from fungus-exposed tumors impaired cytotoxic T-cell killing, increased Treg differentiation, and promoted PD-1 expression in CD8^+^ T cells. Depletion of MDSCs reduced tumor growth and lowered the frequencies of Tregs and PD-1^+^ CD8^+^ T cells (75).

Neutrophil-rich bacterial microniches have also been observed in human tumors. Bacteria-positive regions in OSCC and CRC contained more CD66b^+^ and CD11b^+^ cells than adjacent bacteria-negative regions. Bacteria-positive areas also showed greater ARG1 expression and lower CD4^+^ and CD8^+^ T-cell densities. CXCL8, IL-17C, and other tumor-derived chemotactic signals mediated microbiota-associated neutrophil recruitment in the evaluated tumor models. The functional outcome varied according to tumor model and activating signal, indicating that neutrophil abundance alone does not distinguish antitumor from protumor activity (12, 76).

Neutrophil extracellular trap (NET) formation is relevant only in tumor settings for which direct evidence is available. Protumor neutrophils may release neutrophil elastase and MMP-9, while NET formation has been associated with extracellular matrix remodeling and metastatic dissemination. In microbiota-related models, activated neutrophils formed NET-like structures between tumor cells and NK cells, limiting direct NK–tumor cell contact. These findings do not demonstrate that NETs broadly mediate CD8^+^ T-cell exclusion across tumor types (87, 88).

MDSCs and protumor neutrophils can suppress lymphocytes through overlapping mechanisms. ARG1 reduces extracellular arginine availability, while ROS and nitric oxide interfere with T-cell activation and cytotoxicity. Whether dense neutrophil or MDSC clusters also form a physical barrier that limits effector-cell access to microbe-rich tumor regions remains unresolved. Spatial co-mapping of microbial signals, NET components, myeloid-cell boundaries, and lymphocyte trajectories would be required to distinguish biochemical suppression from physical exclusion (89).

Stromal and Vascular Constraints on Regional Immunity. Direct evidence for a microbiota–fibroblast pathway driving immune exclusion remains limited. In OSCC and CRC, epithelial regions containing bacterial signals showed lower vascular density and reduced α-smooth muscle actin staining than bacteria-negative regions. Lower vascular density does not, by itself, indicate impaired perfusion, increased permeability, or vascular collapse. The reduction in α-smooth muscle actin also cannot be assigned specifically to CAFs because the cellular origin of the signal was not determined (90–92).

Microbial infection can alter tumor-cell interactions with the extracellular matrix and may contribute indirectly to the organization of myeloid-rich niches. *Fusobacterium nucleatum*-infected CRC cells detached from spheroids, migrated individually through collagen, retained intracellular bacteria, and upregulated transcriptional programs related to extracellular-matrix remodeling, adhesion, migration, EGFR, and PDGF signaling. Infected spheroids also recruited and retained neutrophils. These findings support altered tumor-cell behavior at the matrix interface and possible production of myeloid-attracting signals, but they do not demonstrate increased collagen deposition, collagen alignment, or formation of a fibroblast-derived exclusion barrier (12).

Macrophage-derived mediators and extracellular vesicles provide a possible indirect link with the stromal compartment. M2-like TAMs can produce TGF-β, VEGF, PDGF, and MMPs, while vesicles from *F. nucleatum*-infected tumor cells can transfer regulatory molecules to fibroblasts and endothelial cells. Such interactions have been associated with angiogenesis, vascular permeability, lymphangiogenesis, and formation of permissive metastatic niches. Direct spatial evidence linking microbial signals to CAF activation and lymphocyte exclusion remains limited (79, 82).

Regions with a higher intratumoral microbial signal have shown greater myeloid-cell accumulation, lower vascular density, and differences in the expression of matrix-remodeling genes. The available evidence does not demonstrate that intratumoral microorganisms directly organize collagen fibers or generate a continuous CAF-dependent barrier. Further evaluation will require spatial mapping of microbial signals, CAF subpopulations, collagen architecture, blood vessels, and effector lymphocytes within the same tumor specimens (18, 93).

Regional Integration of Myeloid Inflammation and T-Cell Function. Myeloid-dominant inflammation should be distinguished from productive antitumor immunity. Regions enriched in macrophages, neutrophils, MDSCs, or inflammatory mediators may still contain few functional cytotoxic lymphocytes or restrict their access to malignant-cell nests. Assignment of an immune-inflamed state therefore requires lymphocyte entry into malignant-cell regions together with evidence of activation or cytotoxic function, whereas abundant innate inflammation alone may be compatible with immune suppression or exclusion (94).

Microbial stimulation can induce IL-1β, IL-8, IL-17, TNF, ROS, and other inflammatory mediators without producing effective tumor rejection. In experimental colorectal tumorigenesis, microbial products induced inflammatory cytokines followed by PMN-MDSC accumulation, shifting the local immune composition toward suppression. *Fusobacterium*-associated tumors may likewise contain elevated inflammatory mediators, although inflammatory activity alone does not establish productive cytotoxic immunity (78, 95).

The bacteria-positive microniches described above provide a spatial example of myeloid-dominant inflammation associated with reduced local T-cell representation. A comparable suppressive pattern was observed in lung adenocarcinoma, where a low-biomass intratumoral fungus promoted MDSC expansion, arginase and ROS production, and impaired cytotoxic lymphocyte activity. Both settings illustrate that microbial signals can coexist with substantial innate immune activity without generating an effective immune-inflamed regional state (96, 97).

The consequences of microbial inflammation nevertheless remain context-dependent. *F. nucleatum* has been associated with lymphocyte suppression in some tumors but with greater immune-cell recruitment or improved checkpoint-blockade response in others. In gastric cancer, higher *F. nucleatum* loads coincided with increased PD-L1^+^ tumor-associated neutrophils and greater sensitivity to anti-PD-L1 treatment despite evidence of neutrophil-mediated suppression. Microbial burden, inflammatory cytokines, and myeloid-cell abundance should therefore be interpreted together with lymphocyte localization, proliferation, cytotoxic activity, tumor-cell contact, vascular accessibility, and stromal organization. Low CD8^+^ T-cell abundance alone cannot establish immune exclusion; localized comparisons among malignant-cell nests, stromal compartments, and microbe-rich microniches remain necessary (98–100).

### Microbial metabolic activity and functional consequences within tumor niches

Microbial metabolites and local host signaling. Tumor-cell glycolysis is a well-established source of extracellular lactate, whereas the contribution of intratumoral bacteria remains undefined. Lactate accumulation can modify vascular signaling, macrophage states, lymphoid-cell function, and metabolic exchange between hypoxic and oxygenated tumor regions. Bacterial fermentation could add to the local lactate pool, but no spatially resolved analysis has separated microbial production from malignant and stromal sources in human tumors. Assigning a substantial role to microbial lactate will require isotope tracing or equivalent measurements of metabolic flux within bacteria-containing tumor regions (63, 64).

Local microbial activity has been demonstrated more directly for tryptophan metabolism. In melanoma models, *Lactobacillus reuteri* translocated to tumors, persisted in tumor tissue, and released indole-3-aldehyde. The metabolite activated AhR-dependent CREB signaling in CD8^+^ T cells and increased IFN-γ-producing Tc1 responses. Loss of bacterial indole-3-aldehyde production or CD8^+^ T-cell AhR signaling reduced the antitumor effect, whereas dietary tryptophan availability modified treatment response. The melanoma model connects a defined substrate, an intratumoral microorganism, a microbial metabolite, a host receptor, and an immune phenotype within one experimental setting (56, 101). Together with the suppressive myeloid, regulatory, stromal and T-cell states described above, these metabolic and functional outcomes are integrated in **Figure 2C**.

SCFAs require strict source attribution. Effects observed after intestinal microbial manipulation, systemic metabolite administration, or exposure of cancer cells to exogenous SCFAs cannot be assigned automatically to production by tumor-resident microorganisms. Inosine may support activated CD8^+^ T-cell metabolism under glucose restriction and has also been associated with UBA6 inhibition in malignant cells, but its local microbial production within tumor microniches remains unresolved. Demonstration of intratumoral microbial origin would require spatially matched metabolite measurements, evidence of microbial pathway activity, or isotope-based tracing (102, 103).

*Fusobacterium nucleatum-*associated formate has been reported to activate AhR signaling and to increase invasive and stem-like features in CRC models. Local formate production by viable bacteria, however, has not been demonstrated in spatially resolved human tumor tissue. Interpretation of bile acid–related effects is also complicated because several proposed mechanisms involve microbial transformation in the intestinal lumen, followed by systemic circulation or exposure of tumor tissue. Evidence directly linking intratumoral microbial synthesis of polyamines or sphingolipids with defined host responses is currently insufficient. Polyamines and sphingolipids are therefore better considered candidate mediators until microbial origin, local concentration, and host target engagement are demonstrated within tumors (58, 64, 104).

Microbial Reprogramming of Tumor-Cell Metabolism. Several microorganisms have been linked to changes in malignant-cell metabolism. Tumor-resident *Lactobacillus iners* was reported to confer chemoradiation resistance in cervical cancer through lactate-associated metabolic rewiring. In CRC, *F. nucleatum* can induce the long non-coding RNA ENO1-IT1, alter histone regulation, and increase glycolytic activity. Microbial effects may also create metabolic vulnerabilities. *C. butyricum*-associated butyrate increased lipid accumulation and oxidative stress in pancreatic cancer cells and sensitized them to ferroptosis. *Paraburkholderia fungorum* was associated with changes in alanine, aspartate, and glutamate metabolism in intrahepatic cholangiocarcinoma, but enrichment mainly in paracancerous tissue limits interpretation as a confirmed intratumoral interaction (63, 104, 105).

Spatial evidence also links bacterial colonization with reduced malignant-cell proliferation. Bacteria-positive regions showed lower Ki-67 expression, while cell-associated bacterial signals were associated with transcriptional programs related to dormancy and DNA repair. Reduced Ki-67 expression does not establish a metabolic cause. Lower proliferation could reflect hypoxia, nutrient limitation, direct microbial signaling, immune pressure, or combined effects. A microbial contribution to tumor-cell quiescence will remain uncertain until microbial viability, metabolite production, host metabolic flux, and cell-cycle changes are measured in the same spatial compartment (12).

Microbial Modulation of Anticancer Drug Response. Among microbial effects on anticancer drugs, intratumoral evidence is strongest for gemcitabine inactivation. Gammaproteobacteria isolated from pancreatic tumors express a long isoform of cytidine deaminase that converts gemcitabine into inactive 2′,2′-difluorodeoxyuridine, and antibiotic treatment restored responsiveness in an experimental model (106, 107). *Escherichia coli* can also reduce 5-fluorouracil toxicity through the preTA operon, which converts the drug to dihydrofluorouracil. The available evidence does not establish that tumor-resident *E. coli* performs this reaction at biologically relevant rates within human tumor microniches. Enzymatic deamination and reduction represent direct modifications of drug bioavailability, but their anatomical site must be established independently (108, 109).

Microorganisms can also protect malignant cells without directly degrading the drug. *F. nucleatum* activates TLR4–MYD88 signaling, reduces miR-18a* and miR-4802, and increases autophagy, thereby altering responses to 5-fluorouracil and oxaliplatin (110). BIRC3 upregulation provides an additional anti-apoptotic route to 5-fluorouracil resistance (111). Other colorectal tumor-associated bacteria have been reported to reduce 5-fluorouracil bioavailability, although the responsible enzymes remain undefined in several species. Targeted bacterial depletion, including phages directed against *F. nucleatum* or adherent-invasive *E. coli*, has improved treatment responses or reduced tumor burden in experimental CRC models. Intracellular localization and strain-level variation may limit translation (66, 105).

## Tumor-type-specific microbial diversity and its spatial context across solid cancers

The taxonomic breadth of the intratumoral microbiome extends beyond the bacterial species most frequently discussed in cancer, but the spatial resolution of this evidence differs considerably among tumor types. Direct regional comparisons remain concentrated in CRC and OSCC, where microbial signals have been mapped to bacteria-positive and bacteria-negative microniches within the same tissue. In other cancers, bacterial and fungal taxa have been identified through sequencing, cell-resolved analyses, *in situ* detection, cultivation, or experimental perturbation, but their relationship with immune architecture has not consistently been resolved within intact tumor regions. Cross-cancer comparison therefore requires distinction between direct regional localization, cellular compartmentalization, tumor-level association, and experimentally supported mechanism, rather than treating these levels of evidence as equivalent (8, 12, 51).

CRC and head and neck tumors currently provide the clearest direct evidence that microbial enrichment can coincide with spatially restricted immune states. In CRC and oral squamous cell carcinoma (OSCC), bacteria-positive microniches contained greater CD11b^+^ and CD66b^+^ myeloid-cell representation, increased ARG1-associated signals, fewer local CD4^+^ and CD8^+^ T cells, and reduced vascular-associated and proliferative features relative to adjacent bacteria-negative regions. Cell-resolved analysis of OSCC further linked bacterial signals in malignant epithelial cells and monocyte-derived macrophages to reduced T-cell-related transcription and altered cDC1-associated communication. In head and neck squamous cell carcinoma (HNSCC), higher total intratumoral bacterial burden was associated with neutrophil accumulation, depletion of adaptive immune populations, and resistance to PD-L1 blockade; experimental manipulation of bacterial burden reproduced the corresponding immune changes. Regional bacteria-positive versus bacteria-negative comparisons therefore provide direct evidence of spatially restricted microbial–immune organization, whereas tumor-wide bacterial burden in HNSCC provides functional evidence without equivalent microniche resolution (12, 112, 113).

The intratumoral bacterial spectrum extends beyond *Fusobacterium nucleatum* and *Escherichia coli*, although spatial resolution remains uneven across emerging taxa. In CRC models, *Peptostreptococcus anaerobius* promoted CXCL1-dependent recruitment of CXCR2^+^ myeloid-derived suppressor cells and enhanced their suppressive activity through bacterial lytC_22–Slamf4 signaling, but the corresponding organism–MDSC relationship has not yet been mapped within defined human CRC microniches. In gastric cancer, *Streptococcus anginosus* was recovered from tumor tissue and localized by FISH; greater intratumoral abundance was accompanied by lower CD8^+^ T-cell infiltration, while experimental analyses linked tumor promotion to bacterial arginine metabolism and increased ornithine production. Tumor-resident *Lactobacillus iners* in cervical cancer was associated with poor chemoradiation response and promoted resistance through L-lactate-dependent metabolic rewiring of malignant cells. Among the three taxa, *S. anginosus* currently combines direct tissue localization with a tumor-level immune association, whereas the regional organization of the corresponding host responses remains unresolved (114–116).

In gastric cancer, microbial identity has been associated with different myeloid and lymphoid programs. Intratumoral *Fusobacterium nucleatum* was enriched in tumors with lymph-node metastasis and adverse outcome, while experimental infection activated IL-17/NF-κB/RelB signaling and promoted recruitment and protumoral differentiation of PD-L1^+^ tumor-associated neutrophils. The resulting neutrophil program supported immune evasion but also increased sensitivity to PD-L1 blockade. *Ligilactobacillus salivarius* showed an opposing association: it was depleted in gastric tumors but enriched in patients responding to immune-checkpoint blockade, and its bacterial extracellular vesicles activated FPR1-dependent pro-inflammatory macrophage programs that enhanced anti-PD-1 activity in experimental gastric cancer models. At the cohort level, intratumoral microbial associations with CCL4, CCL5, CXCL9, CXCL10, and CXCL11 differed according to prognostic risk and were linked to immune-cell recruitment (117–119).

Pancreatic ductal adenocarcinoma (PDAC) contains both suppressive and immune-associated bacterial configurations. In experimental PDAC, endogenous tumor-associated bacteria promoted suppressive monocytic differentiation through Toll-like-receptor signaling, increased MDSC-associated immune tolerance, and restrained CD4^+^ and CD8^+^ T-cell activation. Microbial depletion shifted macrophages toward an M1-like state, increased Th1 and CD8^+^ T-cell activity, and improved responsiveness to checkpoint-directed therapy. In human PDAC, long-term survivors had greater intratumoral bacterial diversity together with higher CD3^+^, CD8^+^, and granzyme-B^+^ cell densities, while microbiota-transfer experiments altered tumor growth and immune recruitment. An independent human cohort linked intratumoral anaerobic bacteria, including *Bacteroides*, *Lactobacillus*, and *Peptoniphilus*, to lower densities of CD4^+^, CD8^+^, and CD45RO^+^ tumor-infiltrating lymphocytes and poorer prognosis (70, 120, 121).

Intratumoral mycobiome across solid cancers. Spatial characterization of the intratumoral mycobiome remains less developed than taxonomic and functional characterization. Fungal signals have been localized in association with malignant cells and macrophages, but paired mapping of fungal-signal-positive and fungal-signal-negative regions together with their local immune composition remains uncommon. Fungal communities nevertheless differ markedly among tumor types, and fungal–bacterial relationships vary according to tumor origin. *Candida* species are enriched in several gastrointestinal tumors, where viable and transcriptionally active fungi have been recovered; greater *Candida* representation has also been associated with metastatic CRC and reduced survival across gastrointestinal malignancies. In lung adenocarcinoma, *Aspergillus sydowii* was enriched within tumor tissue and associated with greater immunosuppressive myeloid representation and poorer clinical outcome. Low fungal biomass remains an important interpretive constraint because fungal DNA detection alone cannot distinguish viable intratumoral organisms from nonviable or transient microbial material (51, 75, 122).

Fungal species can alter the tumor immune microenvironment through distinct pathways. In PDAC, *Malassezia* accumulated within tumor tissue, and *Malassezia globosa* promoted tumor growth through mannose-binding lectin-dependent complement activation involving mannose-binding lectin, C3, and tumor-cell C3aR. A separate fungal pathway in pancreatic tumors increased cancer-cell IL-33 secretion, followed by recruitment and activation of Th2 cells and group 2 innate lymphoid cells (ILC2s); fungal depletion reduced type 2 immune infiltration and prolonged survival. In lung adenocarcinoma, *Aspergillus sydowii* activated β-glucan–Dectin-1–CARD9 signaling, increased IL-1β production, and expanded suppressive MDSCs with greater arginase, reactive oxygen species, and nitric-oxide-associated activity. The resulting myeloid state impaired cytotoxic T-cell function and was accompanied by greater PD-1^+^ CD8^+^ T-cell representation. Fungal-associated immune remodeling therefore differs among tumor settings, involving complement activation, type 2 immunity, or MDSC-mediated suppression according to the fungal species and host tissue. Direct localization and functional perturbation support tumor-associated fungal localization and functional effects in selected settings, but recurrent fungal-signal-positive immune microniches analogous to the bacteria-positive regions mapped in CRC and OSCC have not yet been established (75, 121, 123).

In breast cancer, cellular localization can change the immune effect of tumor-associated bacteria. Human breast tumors contain intracellular bacterial signals within malignant and immune-cell compartments, while tumor-resident bacteria can persist within malignant cells and accompany metastatic dissemination. Cytosolic bacterial localization induced double-stranded-DNA accumulation and activated a tumor-cell-intrinsic cGAS–STING–IL-17B pathway, generating protumor neutrophils that inhibited cytotoxic T-cell activity. When the same bacterial strain remained extracellular, neutrophils instead acquired antitumor activity without equivalent STING engagement. Intracellular versus extracellular localization therefore changed the host pathway engaged and the functional state of the responding neutrophil population despite equivalent bacterial identity (8, 10, 72).

Cross-cancer comparison does not identify a shared microbial taxon or a fixed microbiota-associated immune state. Microbial signals have been linked to neutrophils, MDSCs, macrophages, dendritic-cell–T-cell communication, type 2 immunity, and metabolic remodeling, with the direction of the association varying according to microbial identity, burden, kingdom, and cellular localization. Tumor-level microbial associations should not be interpreted as evidence of spatial immunomicrobial organization unless microbial and immune features are mapped within the same anatomically registered region. Regional assignment therefore requires localization of the microbial signal, identification of the cellular compartment in which it is detected or encountered, and characterization of the immune populations and pathways present within the same anatomically defined tumor region (8, 72, 122).

## Spatiotemporal remodeling across tumor progression, metastasis, and therapy

Temporal remodeling of the intratumoral microbiome remains less well characterized than spatial heterogeneity. Available studies include cross-sectional comparisons across disease stages, paired primary and metastatic tumors, controlled metastatic models, and repeated biopsies collected during treatment. Stage-based comparisons identify associations with disease severity but cannot reconstruct microbial evolution within an individual lesion. Paired and longitudinal sampling provides stronger support for persistence, replacement, or treatment-associated change. Fecal studies alone cannot establish alterations in microbial burden, localization, viability, or activity within tumor tissue (18, 124, 125).

Premalignant-to-malignant progression. Tumor-tissue studies indicate that microbial composition differs among premalignant lesions, localized tumors, and advanced disease, although most comparisons remain cross-sectional. Colorectal adenomas and carcinomas display distinct microbial profiles together with marked regional heterogeneity within individual lesions. In OSCC, advanced tumors have been associated with greater abundance of *Porphyromonas gingivalis*, while stage-based analyses have reported increasing *Fusobacterium* and decreasing *Streptococcus*, *Haemophilus*, *Porphyromonas*, and *Actinomyces*. Stage-associated variation does not demonstrate that one microbial community developed into another within the same tumor (78, 126).

Comparisons of normal and malignant tissue also suggest microbial selection within the tumor environment. In the MMTV-PyMT breast cancer model, tumors contained an approximately tenfold greater bacterial burden than normal breast tissue, whereas bacterial density remained relatively stable as tumor size increased. Several genera occurred in both normal and malignant breast tissue, consistent with a possible shared tissue source. Similarity between normal-adjacent and tumor-associated communities has also been reported in human cancers, but samples obtained at a single surgical time point cannot resolve whether microorganisms entered the tumor from adjacent tissue, spread from the tumor into surrounding tissue, or arrived from another anatomical site (10, 95).

The driver–passenger model proposes that microorganisms associated with early carcinogenic conditions may later be replaced by taxa better adapted to the established tumor environment. Altered epithelial integrity, oxygen availability, nutrient supply, immune pressure, and tissue metabolism could favor microbial replacement. Current support derives mainly from comparisons among normal tissue, premalignant lesions, and established cancers rather than serial analysis of the same lesion. Microbial succession should remain a working hypothesis until longitudinal tissue sampling, experimental tracing, or strain-resolved analyses demonstrate replacement directly (78, 127).

Primary tumors and metastases. Intracellular bacteria can accompany disseminating tumor cells under controlled experimental conditions. In the MMTV-PyMT breast cancer model, selective depletion of tumor-resident bacteria reduced lung metastasis by more than threefold without a comparable reduction in primary tumor growth. Microbial profiles from primary breast tumors and early pulmonary micrometastases were closely related, whereas established macrometastases showed an intermediate composition between primary tumors and normal lung tissue. The progression from early to established metastasis was also accompanied by an increase in aerobic bacteria and a decrease in facultative anaerobes, indicating adaptation to the pulmonary environment (72, 128, 129).

Metastatic lesions showed greater bacterial abundance, and bacterial signals were detected more frequently in circulating tumor-cell clusters than in single circulating tumor cells. In a tracing experiment, recombinant *Staphylococcus xylosus* introduced into primary tumors was later recovered from lung metastases. Intravenous administration of *S. xylosus* alone did not result in comparable pulmonary detection, whereas bacteria carried by tumor cells reached metastatic sites. The effect on metastatic outgrowth also varied among the tested *Staphylococcus* and *Lactobacillus* strains, suggesting that bacterial carriage alone was insufficient to explain the phenotype (10).

Human tissue studies support microbial continuity between primary and metastatic sites but remain less conclusive regarding transmission. In breast cancer, microbial communities from lymph-node metastases clustered closely with paired primary tumors. Correlated abundance of *Fusobacterium* and co-occurring taxa has also been reported between primary colorectal tumors and liver metastases. Community-level or genus-level similarity is compatible with persistence or cotransport, but it cannot establish that identical strains moved from the primary lesion to the metastatic site (126, 130, 131).

Local conditions at the metastatic site also shape microbial composition. A pan-cancer analysis of 4,160 metastatic biopsies found that the anatomical location of the sampled lesion explained microbial community variation more strongly than the tissue of origin of the primary cancer. Richness and community structure differed among metastatic organs, and anaerobic taxa were enriched in tumors with higher hypoxia scores. The association between hypoxia and anaerobic enrichment does not determine whether bacteria contributed to low oxygen conditions or preferentially colonized lesions that were already hypoxic (14).

Assessment of persistence requires the highest taxonomic resolution permitted by the data. Tumor-derived microbial genomes assembled from repeated metastatic biopsies showed greater community and strain-level similarity within matched samples than among unrelated tumors. The result supports partial microbial persistence over time. Persistence across metastatic biopsies is nevertheless distinct from transmission between a primary tumor and a metastasis. Demonstration of transmission requires paired sampling of both sites together with strain-level genomic identity or direct experimental tracing (14, 132).

Treatment-induced remodeling. Treatment-associated remodeling has been examined most directly in paired metastatic biopsies. Bacterial richness decreased significantly after immune-checkpoint blockade, whereas no overall change was detected after targeted or hormonal treatment. Chemotherapeutic agents with recognized antimicrobial activity, including anthracyclines and bleomycin, may additionally influence microbial communities during treatment, although the observed reduction in tumor-associated richness was less consistent than that following checkpoint blockade (14, 112, 133).

Among patients receiving checkpoint blockade, richness reduction was more evident in patients with stable disease or partial response than in those with progressive disease. Actinomyces, Bacteroidetes, Bifidobacterium, and Prevotella were among the microbial groups recurrently depleted in patients with disease control. The direction of the relationship remains unresolved. Microbial loss could facilitate immune activation, result from treatment-induced immune pressure, or accompany tumor regression without contributing directly to it (14, 134).

Pretreatment microbial composition may also carry predictive information. In metastatic NSCLC, greater *Fusobacterium* abundance before checkpoint blockade was associated with reduced durable clinical benefit, shorter survival, and lower cytotoxic, IFN-γ, and MHC class II transcriptional signatures. Baseline *Fusobacterium* abundance was associated with subsequent treatment outcomes. The role of Fusobacterium in shaping immune responses during checkpoint blockade was not examined, and the therapeutic effect of its depletion remains unknown (14, 135).

Most chemotherapy and radiotherapy studies have examined microbial effects on treatment efficacy rather than treatment-induced remodeling of the intratumoral microbiome. Defined microbial mechanisms can alter responses to gemcitabine, fluoropyrimidines, oxaliplatin, or chemoradiation, but the available studies do not establish whether these treatments modify intratumoral microbial burden, localization, viability, or transcriptional activity. Evidence that microorganisms affect treatment response should therefore not be interpreted as evidence that treatment remodels the intratumoral microbiome (95, 102).

Antibiotic experiments offer more direct evidence of microbial perturbation when local and intestinal effects are separated. In the MMTV-PyMT model, intravenous or cell-penetrating antibiotics reduced tumor-resident bacteria while largely preserving the intestinal community. Tumor-local depletion decreased lung metastasis without reducing primary tumor mass, supporting a local microbial contribution to metastatic colonization in that experimental setting (10).

Systemic and orally administered broad-spectrum antibiotics do not isolate intratumoral effects from changes in the intestinal microbiota and host immunity. Reduced checkpoint-inhibitor efficacy after antibiotic exposure cannot be attributed specifically to depletion of the tumor microbiome. Tumor-directed or nanoparticle-delivered antibiotics have reduced metastasis or altered immune infiltration in selected preclinical models while limiting disruption of intestinal communities. Route of administration, tissue penetration, antimicrobial spectrum, treatment timing, and effects on non-tumor microbial compartments should be reported in studies of antibiotic-associated remodeling (95, 127, 136).

Most studies of surgically resected tumors have examined whether intratumoral taxa are associated with recurrence or survival. Reports in colorectal, lung, gastric, and pancreatic cancer generally rely on tissue collected at resection rather than longitudinal sampling of the same lesion. Such datasets cannot establish whether surgery alters the intratumoral microbiome. Direct evaluation would require pretreatment biopsies matched to the resected tumor, or paired primary and recurrent tumors, with perioperative antibiotic exposure and intervening therapies recorded as potential confounders (78, 137, 138).

Longitudinal evidence and paired sampling. The strongest longitudinal human evidence comes from a metastatic cohort containing 185 biopsy pairs from 173 patients, with a median interval of 7.5 months. Bacterial abundance was moderately correlated between time points, and repeated biopsies from the same lesion were more similar than randomly paired tumors. Strain-level comparisons also showed greater genomic similarity within matched samples. Partial persistence was therefore detectable despite compositional change during disease progression and treatment (14).

Interpretation depends on the anatomical relationship between paired specimens. Repeated biopsies from the same lesion are best suited to evaluating temporal persistence, whereas samples from different metastatic organs combine temporal change with organ-specific selection. Analyses should account for the interval between biopsies, lesion location, tumor purity, cancer type, treatment exposure, and whether the sampled lesion was progressing or responding. Several of those variables were incorporated into the paired models of the metastatic cohort, reducing but not eliminating residual confounding (139, 140).

The sequence of normal breast, primary tumor, early pulmonary micrometastasis, and established lung metastasis examined in an experimental breast cancer model provides controlled information across metastatic progression. Bacterial cotransport was followed by compositional adaptation within the metastatic organ. The samples represent different stages of an experimental metastatic process rather than serial biopsies of the same human lesion and should not be interpreted as direct evidence of natural longitudinal evolution in patients (10).

Paired primary-recurrent tumors, serial metastatic biopsies, and repeated spatial sampling remain uncommon in the available literature. Longitudinal studies should record the exact lesion sampled at each time point, treatment and antibiotic exposure between collections, sampling depth, tumor cellularity, microbial viability, and contamination controls. Strain-resolved sequencing should be combined with localization methods and measurements of microbial transcription or metabolism when persistence and functional adaptation are being evaluated (141, 142).

Transitions among immune states. The bacteria-positive and bacteria-negative microniches described above demonstrate that contrasting immune–microbial conditions can coexist within the same tumor. However, their cross-sectional characterization cannot determine whether an immune-inflamed region subsequently became immune-excluded, immune-suppressed, or immune-desert, or acquired a more exhausted T-cell phenotype. Longitudinal spatial sampling of the same lesion would be required to demonstrate a transition between regional immune ecotypes (12).

Intratumoral microorganisms can be associated with either immune activation or immune suppression. Some taxa and microbial metabolites promote IFN-γ-producing CD8^+^ T cells, dendritic-cell activation, or inflammatory macrophage polarization. Others coincide with increased PD-1, LAG-3, and TIGIT expression, reduced cytotoxic-cell infiltration, M2 macrophages, MDSCs, and suppressive neutrophils. Persistent exposure to microbial antigens may favor T-cell exhaustion, although support for this mechanism comes mainly from experimental and cross-sectional studies. A shift from an inflamed to an exhausted state has not been documented longitudinally within the same human tumor (80).

Checkpoint blockade was followed by lower microbial richness in paired metastatic biopsies, particularly among patients with disease control. Paired spatial profiling of the microbiome and immune compartment was not performed, so the microbial change cannot be assigned to an excluded-to-inflamed, inflamed-to-exhausted, or inflamed-to-desert transition. The association between pretreatment *Fusobacterium* abundance and reduced cytotoxic immune signatures likewise reflects differences among tumors rather than temporal conversion within the same lesion (143–145).

Engineered *Lactococcus lactis* and *Escherichia coli* increased dendritic-cell infiltration and CD8^+^ T-cell activation in mouse tumor models, generating an immune profile consistent with cold-to-hot conversion. Comparable spontaneous transitions have not been demonstrated in patients, and cross-sectional single-cell or spatial data cannot determine whether microbial changes precede or follow immune-state remodeling (80, 146).

## Spatial technologies, multi-omic integration, causal validation, and microbiome-guided tumor reprogramming

Spatial detection and multi-omic integration. Characterization of spatial microbial–immune configurations requires methods that resolve several non-equivalent variables: the anatomical position of a microbial signal, its assignment to an extracellular or host-cell compartment, the identity and functional state of surrounding host cells, and variation among regions of the same lesion. No single platform simultaneously provides broad microbial taxonomy, cellular localization, microbial viability, host molecular profiling, and tissue-scale architecture. Direct microbial imaging, spatial host–microbiome sequencing, host-focused spatial profiling, broad microbial sequencing, and functional assays therefore provide complementary levels of evidence. Human spatial observations and experimental mechanistic evidence are summarized separately in **Table 1** to distinguish regional microbial–immune associations from causal effects demonstrated in experimental models (148–150).

**Table 1:** Human spatial and experimental evidence linking intratumoral microbial signals to regional tumor immune ecotypes.

Regional immune state	Defining host spatial feature	Human spatial evidence and tumor type	Experimental evidence and model	Evidence level	References
Immune-inflamed	Cytotoxic lymphocytes within or immediately adjacent to malignant-cell nests, with local IFN-γ-associated activity.	Not directly established. In human melanoma and glioblastoma, bacteria-derived HLA-bound peptides and microbial-peptide recognition by tumor-infiltrating lymphocytes demonstrate microbial antigen presentation, but do not spatially map microbial signals together with an immune-inflamed regional ecotype.	Murine melanoma: intratumoral *Lactobacillus reuteri*-derived indole-3-aldehyde enhanced AhR-dependent IFN-γ-producing CD8+ T-cell responses.	Experimental support; no direct human spatial evidence.	(11, 56, 147)
Immune-excluded	Lymphocytes accumulate at the invasive margin or in stromal compartments but have limited access to malignant-cell nests.	CRC and OSCC: bacteria-positive microniches contained fewer local CD4+ and CD8+ T cells and lower vascular-associated signals than adjacent bacteria-negative regions. Direct microbiota-associated stromal retention or exclusion from malignant-cell nests was not demonstrated.	No experimental model cited here establishes formation of a continuous microbiota-dependent CAF- or collagen-associated barrier responsible for T-cell exclusion.	Partial human spatial evidence.	(2, 12, 19)
Immune-desert	Low effector-cell recruitment in both stromal and malignant-cell compartments.	No direct human spatial evidence has established a reproducible microbial configuration associated with immune-desert regions.	No experimental evidence currently establishes microbiota-driven formation of a regional immune-desert ecotype.	No direct spatial evidence.	(1, 3)
Immune-suppressed	Immune-infiltrated region with restricted cytotoxic activity and/or dominance of suppressive myeloid or regulatory-cell programs.	CRC and OSCC: bacteria-positive microniches showed increased CD11b+ and CD66b+ myeloid-cell representation and ARG1-associated signals together with reduced local CD4+ and CD8+ T-cell representation.	Lung adenocarcinoma models: intratumoral *Aspergillus sydowii* promoted MDSC-mediated suppression. HNSCC models: increased intratumoral bacterial burden promoted neutrophil-dominant immunosuppression and resistance to PD-L1 blockade.	Direct human spatial association with experimental mechanistic support.	(12, 75, 113)
Intralesional coexistence of regional immune states	Distinct immune ecotypes occupy spatially separate regions within the same lesion.	CRC and OSCC: adjacent bacteria-positive and bacteria-negative territories showed distinct cellular and transcriptional configurations. Independent human spatial studies demonstrate coexistence of distinct immune neighborhoods within individual tumors.	Experimental evidence is not required to establish spatial coexistence; temporal transitions among regional ecotypes remain unproven.	Direct human spatial evidence of regional heterogeneity.	(2, 4, 12)

FISH and RNAscope provide direct *in situ* localization of microbial nucleic acids and can distinguish focal from diffuse distribution within intact tissue. Pan-bacterial probes establish broad bacterial localization, whereas validated taxon-specific probes can resolve selected genera or species. Combination with epithelial, immune, stromal, or vascular markers allows microbial signals to be registered against defined host compartments. Optical sectioning, three-dimensional imaging, and, where required, electron microscopy provide stronger evidence for intracellular localization than two-dimensional signal overlap alone. Probe specificity and limited taxonomic breadth remain important constraints, and detection of microbial RNA or morphology does not independently establish replication or sustained viability (12, 151).

Sequencing-based spatial approaches extend localization beyond predefined microbial probes. Spatial transcriptomic analysis in human OSCC and CRC recovered bacterial transcripts together with anatomically registered host transcriptional profiles, permitting comparison of bacteria-positive and bacteria-negative regions. Spatial host–microbiome sequencing (SHM-seq) combines tissue histology, host transcriptomics, and bacterial 16S capture on the same spatially barcoded section, while spatial metatranscriptomics (SmT) captures host transcripts together with bacterial 16S and fungal 18S/ITS signals. SHM-seq was demonstrated in the mouse gut and SmT in *Arabidopsis thaliana* tissue; neither has yet become a routine method for low-biomass human tumors. Translation to cancer tissue will require adequate microbial read depth, stringent contamination control, and orthogonal confirmation of microbial localization (12, 149, 152).

Host-focused spatial profiling addresses the cellular environment surrounding a microbial signal rather than microbial identity itself. GeoMx digital spatial profiling can quantify proteins or transcripts within anatomically defined regions, while multiplex and high-plex imaging can resolve cellular composition, stromal organization, vascular structures, and cell–cell relationships. In OSCC and CRC, RNAscope was used to define bacteria-positive and bacteria-negative regions before comparison of their epithelial and immune features using GeoMx and immunohistochemistry. Such approaches support regional microbial–host analysis only when microbial and host measurements remain anatomically registered; host spatial architecture alone does not establish the presence of microorganisms (12, 45).

Cell-resolved microbial approaches provide host-cell assignment without preserving the complete tissue architecture. INVADEseq links bacterial 16S rRNA reads to individual host-cell barcodes and can identify transcriptional programs associated with microbial signals, whereas SAHMI and CSI-Microbes recover candidate microbial reads from conventional single-cell RNA-sequencing datasets. Tissue dissociation removes the original anatomical coordinates of the analyzed cells, while ambient RNA, sparse microbial reads, host-sequence misclassification, and surface-associated microorganisms complicate interpretation. Cell-associated microbial signals should therefore not be treated as direct evidence of regional colocalization in intact tissue (12, 77, 80).

Conventional 16S sequencing, shotgun metagenomics, and bulk metatranscriptomics answer complementary but non-spatial questions. Shotgun metagenomics provides broader taxonomic and functional resolution, while metatranscriptomics identifies expressed microbial RNA; neither approach, when applied to bulk tumor tissue, determines whether a signal originates from malignant-cell nests, stromal interfaces, vascular regions, mucosal biofilms, or discrete microbial microniches. Such measurements can identify candidate taxa, pathways, or host associations for subsequent spatial testing but do not independently demonstrate regional immunomicrobial organization (153–155).

A complementary route to clinical scalability is to use high-plex spatial profiling as a discovery layer and routine histopathology as a tissue-wide inference layer. In non-small cell lung cancer, 41-plex CODEX profiling identified recurrent tumor, immune, stromal, and vascular neighborhoods that were subsequently used to develop CANVAS, an H&E-based approach for inference of spatial ecological habitats across large patient cohorts. Although not developed to detect intratumoral microorganisms, such host-architecture maps could guide targeted sampling of regions selected for FISH, spatial transcriptomics, spatial proteomics, or metabolomics. H&E-derived or computationally inferred habitats do not measure microbial burden, viability, or function and cannot replace direct microbial localization and orthogonal validation (45).

Quality control and causal validation. Low microbial biomass makes contamination control a central requirement. Microbial material may enter during surgery, transport, fixation, sectioning, extraction, library preparation, or sequencing. Extraction blanks, no-template controls, reagent controls, and environmental controls should accompany tumor specimens throughout the analytical workflow. Computational approaches such as Decontam and SCRuB can support contaminant identification but cannot replace experimental controls, stringent host-read removal, curated reference databases, and inspection of alignment quality (66, 155).

Sequencing-based microbial signals require confirmation by an independent detection principle when biological interpretation depends on localization or intracellular residence. Taxon-specific FISH, RNAscope, quantitative PCR, droplet digital PCR, and electron microscopy provide different forms of orthogonal validation. Intracellular localization requires optical sectioning or three-dimensional imaging and, where feasible, ultrastructural confirmation or methods capable of distinguishing internalized microorganisms from surface-associated material (156, 157).

Microbial viability requires a separate level of evidence. Culture, transcriptional activity, metabolic labeling, and viability-sensitive measurements address different aspects of microbial viability and should not be considered interchangeable. Species- or strain-level resolution becomes particularly important when interpretation depends on cellular invasion, toxin production, antigen presentation, drug metabolism, or susceptibility to antimicrobial intervention (158, 159).

Detection, localization, association, functional interaction, and causality should remain analytically distinct. Detection establishes microbial material; localization assigns the signal to a tissue or cellular compartment; association links microbial features with a host state without demonstrating biological activity. Functional evidence requires a measurable host response, whereas causal inference requires experimental manipulation of the microorganism, its product, or the affected host pathway. Associations with prognosis or treatment response require independent validation before clinical interpretation (18, 160).

Selected experimental systems provide causal evidence for specific microbial effects. Tumor-resident intracellular bacteria increased metastatic fitness in breast-cancer models, while intratumoral *Escherichia coli* MG1655 altered vascular organization, macrophage signaling, CCL5 production, and T-cell recruitment. Experimental depletion, reintroduction, macrophage depletion, or pathway interference supported causal relationships in these models, but the findings do not establish microbiome-driven reprogramming of immune-excluded or cold human tumors (10, 161).

Spatial immunomicrobial biomarkers. Candidate spatial biomarkers should incorporate microbial burden, anatomical location, intracellular or extracellular distribution, and relationships with malignant, immune, stromal, and vascular compartments. Tumor core, invasive margin, vascular or hypoxic regions, stromal interfaces, and lymphoid-rich areas may contain different microbial–host configurations. Host immune states should be assigned from regional immune architecture rather than microbial abundance alone. No microbial classifier capable of distinguishing immune-inflamed, immune-excluded, immune-desert, and immune-suppressed regions has undergone independent validation (77, 162, 163).

Microbe–host distance is interpretable only in conjunction with local cell density, lymphocyte phenotype, microbial identity, vascularization, and tissue architecture. Close proximity may reflect microbial uptake, antigen presentation, inflammatory recruitment, or ineffective antibacterial activation, whereas spatial separation may occur in poorly vascularized or myeloid-rich regions. Bacteria-positive microniches in OSCC and CRC illustrate the analytical potential of such measurements, although temporal order and causality remain unresolved (12, 164, 165).

Intracellular and extracellular microbial signals should be recorded separately because their biological effects and therapeutic accessibility may differ. Intracellular bacteria can influence malignant-cell signaling, antigen presentation, mechanical stress responses, or drug metabolism, whereas extracellular microorganisms may form aggregates, release diffusible products, or interact with surrounding host cells. HLA-bound microbial peptides and spatially localized metabolites add functional information, but neither identifies a regional microbial mechanism without appropriate source attribution and anatomical registration (155, 166, 167).

Combined microbial–immune–stromal profiles may ultimately prove more informative than isolated taxa because the same microorganism can occur within different cellular and metabolic environments. No combined spatial microbial–immune profile has been prospectively validated for treatment response, recurrence, or survival. Such profiles should therefore remain investigational units for spatial stratification rather than clinically established biomarkers (168–170).

Microbiome-guided tumor reprogramming. Microbiome-directed intervention should account for microbial location, function, and accessibility. Intracellular microorganisms, extracellular aggregates, and diffusible microbial products present different therapeutic targets, while modification of the intestinal microbiota cannot be assumed to alter an established intratumoral microbial niche. Engineered bacteria can preferentially occupy selected tumor environments and deliver immunomodulatory molecules; intratumoral *Escherichia coli* MG1655, for example, altered macrophage phenotype, CCL5 production, vascular organization, and T-cell recruitment in experimental tumors. Comparable reproducible conversion of immune-excluded or cold human tumors toward an immune-inflamed state has not been established (16, 155, 161).

Selective microbial removal or functional inhibition may be preferable when a defined organism contributes to immune suppression, drug metabolism, or metastatic behavior. Bacteriophages provide greater taxonomic selectivity than broad-spectrum antibiotics but face limitations related to strain specificity, resistance, tissue penetration, and intracellular accessibility. Microbial enzymes provide another target; intratumoral Gammaproteobacteria can, for example, inactivate gemcitabine through bacterial cytidine deaminase. Therapeutic selection therefore requires confirmation of the relevant microorganism, its spatial accessibility, and its functional activity within the tumor (171–173).

Clinical translation requires evidence that alteration of a microbial target changes the corresponding regional host phenotype. A reduction in microbial burden alone would not demonstrate tumor reprogramming without accompanying changes in CD8^+^ T-cell distribution, suppressive myeloid populations, stromal organization, local antigen presentation, or microbial metabolite activity. Dose, persistence, biodistribution, antimicrobial susceptibility, systemic dissemination, and off-target colonization also require prospective evaluation (174–176). Fecal microbiota transplantation (FMT) and conventional probiotics primarily modify the intestinal microbiota and should not be described as direct intratumoral interventions without paired evidence from tumor tissue. More broadly, precision-microbiome strategies require standardized protocols, validated biomarkers, durable biological effects, and long-term safety before clinical implementation (177).

## Conclusions and future perspectives

A single immune-state label cannot describe the regional organization of a tumor, nor does intratumoral microbial presence have a uniform immunological meaning across solid cancers. The central synthesis of the review is the interpretation of spatial microbial signals against regional immune ecotypes defined independently from microbial detection. Spatially resolved studies in CRC and OSCC show that bacteria-rich microniches can coexist with bacteria-negative regions that differ in vascularization, myeloid-cell representation, T-cell density, checkpoint expression, and malignant-cell transcriptional programs. Evidence from gastric, pancreatic, lung, breast, and head and neck cancers extends the underlying principle beyond these spatially resolved settings: microbial associations with tumor immunity vary according to tumor type, microbial identity and burden, microbial kingdom, and the cellular compartment in which microbial signals are encountered. Intratumoral microbiome–immune relationships should therefore be interpreted within both regional and tumor-specific contexts rather than inferred from tumor-wide microbial abundance or extrapolated from a single cancer type. Current evidence does not establish that each immune ecotype has a characteristic microbial configuration or that comparable microbial signals generate equivalent immune states across different tumors (8, 12, 51).

The biological significance of an intratumoral microbial signal depends on the form of evidence supporting it. In low-biomass tumors, DNA-based detection may represent viable microorganisms, nonviable cells, extracellular material, or contamination introduced during tissue collection and processing. Confidence in biological interpretation increases when taxonomic detection is integrated with appropriate negative controls, *in situ* localization, RNA- or viability-sensitive measurements, and microbial isolation when technically feasible. Host-cell assignment provides an additional level of resolution because microbial material localized within malignant cells may have different biological implications from material internalized or retained by myeloid populations (105, 161).

Inflammatory activity does not independently define the direction or efficacy of an antitumor response. Intratumoral microorganisms may participate in STING-dependent signaling, dendritic-cell recruitment, lymphoid organization, and cytotoxic lymphocyte responses, whereas other microbial settings are associated with neutrophil accumulation, regulatory T-cell expansion, suppressive macrophages, MDSCs, or T-cell dysfunction. The resulting phenotype depends on microbial identity and burden, tumor lineage, host immune state, treatment exposure, and molecular context. In HNSCC, bacterial burden has been associated with checkpoint-blockade response, whereas experimental increases in bacterial burden promoted neutrophil accumulation, T-cell depletion, and resistance to anti-PD-L1 therapy. In this setting, bacterial burden therefore appears to influence treatment response through a neutrophil-dominant suppressive program rather than through taxon-specific effects alone (102, 113, 178).

Taxonomic composition provides only one component of intratumoral microbial function. Genes associated with metabolite production require pathway expression, appropriate substrate availability, metabolite release, and exposure of neighboring host cells to produce a local biological effect (179). Hypoxia, nutrient limitation, treatment pressure, and intracellular or extracellular residence may further modify microbial metabolism across tumor regions. Mechanistic support is strongest when microbial transcription, local metabolite concentrations, spatial colocalization, and activation of the corresponding host pathway are resolved within the same clinical specimen or experimental model. In their absence, taxonomic or genomic data identify metabolic potential rather than directly measured local pathway activity or metabolic flux (14, 80, 105).

The temporal sequence of microbial and immune changes remains unresolved in most human tumors. Cross-sectional specimens cannot distinguish microorganisms involved in early immune remodeling from populations that expand after the development of hypoxia, tissue disruption, metastatic dissemination, or treatment-induced selection. Serial biopsies of metastatic lesions have identified microbial changes during systemic treatment, although their origin and functional relevance remain uncertain. Multiregional sampling before and during treatment could determine whether changes in microbial burden or composition precede, accompany, or follow regional immune reorganization and distinguish persistent microbial residence from transient exposure (14, 78, 167).

Spatial colocalization defines candidate microbial–host relationships that require functional testing for mechanistic attribution. Spatial transcriptomics, FISH, RNAscope, and microbe-anchored digital spatial profiling can relate microbial signals to host features within anatomically registered regions, whereas cell-resolved approaches such as INVADEseq identify microbial–host cell associations after tissue dissociation and therefore do not preserve the original tissue coordinates. Regional and cell-resolved approaches therefore provide complementary evidence, whereas causal attribution requires controlled manipulation of the microorganism, its burden or viability, a microbial product, or the affected host pathway, followed by reproduction or reversal of the predicted immune or malignant-cell phenotype. Microbial biomarkers require comparable rigor because associations with treatment response may vary according to assay performance, biopsy location, tumor type, and regional immune architecture. Independent validation in prospectively collected cohorts is therefore required before clinical generalization (80, 113).

Available findings narrow the therapeutic question from modifying the entire microbiome to targeting a defined microbial function within a spatially delimited tumor microniche. Broad-spectrum antibiotics may reduce immunosuppressive bacterial burdens in selected tumors, but their effects depend on tumor type, antimicrobial exposure, treatment timing, and disruption of the gut–immune axis (102, 113). Bacteriophages, targeted antimicrobials, and engineered bacteria may provide greater specificity, although delivery into restricted tumor niches, persistence, resistance, and off-target effects remain unresolved. A defensible therapeutic target would be a viable or transcriptionally active microbial population present at sufficient abundance, spatially linked to a defined adverse host program, and experimentally shown to contribute to that program. Without evidence connecting microbial detection and localization with a defined host effect and causal perturbation, microbial depletion or enrichment should not be assumed to reconfigure the immune state of an entire lesion. Therapeutic effects may instead remain restricted to particular regional immune ecotypes rather than generating a uniformly inflamed and treatment-responsive tumor phenotype (16).
